# Impact on Quality of Life and Psychological Dimensions in Caregivers of Melanoma and Sarcoma Patients: A Scoping Review

**DOI:** 10.3390/cancers18050809

**Published:** 2026-03-02

**Authors:** Klodjana Lleshi, Malihe Shams, Eleonora Bergo, Marco Pluti, Simone Mocellin, Paolo del Fiore, Alessandra Feltrin

**Affiliations:** 1 Hospital Psychology, Veneto Institute of Oncology IOV-IRCCS, 35128 Padova, Italy; klodjana.lleshi@iov.veneto.it (K.L.); malihe.shams@iov.veneto.it (M.S.); marco.pluti@gmail.com (M.P.); alessandra.feltrin@iov.veneto.it (A.F.); 2 Soft-Tissue, Peritoneum and Melanoma Surgical Oncology Unit, Veneto Institute of Oncology IOV-IRCCS, 35128 Padua, Italy; simone.mocellin@iov.veneto.it (S.M.); paolo.delfiore@iov.veneto.it (P.d.F.); 3 Department of Surgery, Oncology and Gastroenterology—DISCOG, University of Padua, 35122 Padua, Italy

**Keywords:** melanoma, sarcoma, caregiver, psychological dimensions, quality of life

## Abstract

**Background/Objectives**: Although they differ in terms of epidemiological incidence, melanoma and sarcoma are rare, aggressive cancers with a substantial impact on patients’ quality of life and that of their caregivers. Caregivers, who are often family members, experience high levels of stress, anxiety, and caregiving burden, leading to significant psychological, social, and economic repercussions. The aim of this scoping review was to identify the assessment tools used to study the quality of life and psychological well-being of caregivers of patients with melanoma and/or sarcoma, and to highlight the main areas of difficulty. **Methods**: A systematic search of PubMed, Embase, and PsycINFO databases identified 325 studies, of which 16 met the inclusion criteria. Eligible studies were English language publications focusing on caregivers of adult patients (>18 years) with melanoma or sarcoma. Reviews, case reports, animal studies, or clinical trials, abstracts, books or book chapters, and studies without clear information on assessment tools or involving the pediatric population were excluded. **Results**: The studies included (2007–2024) involve 3464 caregivers in all, 211 of whom were caring for patients with melanoma or sarcoma. Caregivers were predominantly female (women-to-men ratio of 2:1) with an average age of 50 years. Caregivers presented severely impaired quality of life, high psychological distress, economic and work difficulties, and limited access to emotional support. **Conclusions**: Cancer caregiving emerges as a complex process influenced by individual, relational, and contextual factors that affect psychological well-being. Despite limitations related to the small number of studies, the predominance of melanoma-focused research, limited sample diversity, and heterogeneous designs and assessment tools, this review highlights the need for a theoretically grounded and clinically integrated approach that recognizes caregivers as active yet vulnerable participants within the care system.

## Simple Summary

Melanoma and sarcoma are rare, aggressive cancers that severely impact both patients and their caregivers, often family members, who experience high stress, anxiety, and caregiving burden. This review examined tools used to assess caregivers’ quality of life and psychological well-being, highlighting the main areas of difficulty. A systematic search identified 16 relevant studies (2007–2024) involving 3464 caregivers, 211 of whom cared for melanoma or sarcoma patients. These caregivers were predominantly female and around 50 years old. Caregivers experienced impaired quality of life, psychological distress, economic and work difficulties, and limited emotional support. The findings emphasize the need for integrated, theory-driven approaches that acknowledge caregivers as vulnerable, active participants in care.

## 1. Introduction

Sarcomas and melanomas are distinct cancers, often characterized by marked clinical aggressiveness, unpredictable courses, and a significant impact on patients’ quality of life [1]. Sarcomas are rare malignant tumors that develop in soft tissues and skeletal muscles. They have numerous subtypes, which necessitate multimodal treatment [1]. Melanomas are malignant tumors that originate from the abnormal proliferation of melanocytes. In advanced forms, they require intensive treatment that presents a high incidence of side effects, necessitating continuous monitoring and a strong support network [2]. Despite their markedly different epidemiological incidence, sarcomas are classified as rare cancers, with an estimated incidence of 3–5 cases per 100,000 people according to the European Society for Medical Oncology (ESMO) guidelines, whereas melanoma is a much more common malignancy. Melanoma incidence varies widely between regions, ranging from approximately 37 cases per 100,000 in Australia and 31 per 100,000 in Denmark and Norway, to around 11 per 100,000 in Southern Europe [3]. This is not due to epidemiological similarity, but rather to their shared characteristics, such as diagnostic complexity, aggressive clinical behavior, the need for highly specialized, multidisciplinary management, and centralized care pathways involving oncology, surgery, pathology, dermatology, and supportive care disciplines. Moreover, both malignancies increasingly involve the use of advanced systemic treatments, including immunotherapy and targeted therapies, which require specific expertise and are associated with complex and sometimes overlapping toxicity profiles. In the ESMO guidelines, cutaneous melanoma is discussed within the broader framework of sarcoma and rare cancers, reflecting shared challenges related to biological heterogeneity, limited responsiveness to conventional chemotherapy, and the need for highly specialized, multidisciplinary management at reference centers [4,5,6]. The authors’ clinical experience and the specific cancer care setting in which these patients are commonly treated also guided this focus. Direct clinical observation within oncology surgery units highlighted the complexity of care pathways and the emotional and support needs of informal caregivers. This supported the decision to focus on this clinical setting when mapping the existing evidence.

In both cases, the chronic and potentially debilitating nature of the disease requires the direct and constant involvement of caregivers, typically family members, in the treatment and care process. The presence of a rare and aggressive tumor has a major impact on the patient’s life and creates a significant psychological, social, physical, and practical burden for informal caregivers. Care does not consist solely of physical support, which is often essential, but also includes symptom management, care organization, and emotional support [7].

Caregivers play a central role in both the daily management of the disease and navigation of practical challenges, such as arranging home care and medical appointments, as well as providing emotional support. This is particularly important given the prognostic uncertainty and limited access to specific information resources associated with the condition. Existing literature widely documents that caregivers of cancer patients experience clinically significant levels of psychological distress, impaired quality of life, and affective symptoms such as anxiety and depression. Previous studies found that over 50% of caregivers experience severe psychological distress, with care needs often unidentified and unmanaged in the clinical setting [7]. The intertwining of the caregiver’s care and emotional burdens has cross-cutting, multidimensional implications for their quality of life.

A recent study found that caregivers experienced a major reduction in their psychological well-being, particularly in their mental health, vitality, and social functioning [8]. These results suggest that caregivers’ quality of life is significantly affected, with negative implications for their psychological well-being and crucial areas such as family relationships and work.

The patient’s clinical and symptomatic status was closely related to caregivers’ perceived burden. Another study reported that an increase in caregiver burden and psychological distress was significantly correlated with the physical and psychological symptoms of cancer patients, particularly depression, fatigue, and pain [9].

Although the role of informal caregivers is essential in cancer management, there is no doubt that it is associated with high psychological vulnerability and a substantial impact on quality of life. The early identification of risk factors and the development of specific psychoeducational and support interventions are strategic priorities for person-centered cancer care.

In light of these considerations, the aim of this scoping review was to map the existing literature on the experiences of informal caregivers of patients with melanoma and/or sarcoma. Using the PCC (Population, Concept, Context) framework, the Population comprised informal caregivers of adults diagnosed with melanoma or sarcoma; the Concept focused on quality-of-life outcomes, psychological distress, and related caregiving challenges; and the Context encompassed any healthcare or home care setting in which caregiving occurred. The main objectives were to identify the tools and methods used to assess caregiver well-being, to explore which aspects of caregiving are associated with higher burden or distress, and to highlight gaps in the literature to guide future research. Quality of life and Psychological dimensions are used as umbrella terms to encompass the multidimensional aspects of caregiver well-being, including psychological, emotional, social, functional, and role-related impacts. A better understanding of these dynamics is essential for providing integrated, patient- and caregiver-centered care.

We chose a scoping review approach because it allows for a comprehensive mapping of the available evidence on this topic, identifying key concepts, assessment tools, and knowledge gaps, which is particularly appropriate given the heterogeneous and limited literature on caregivers of patients with melanoma and sarcoma.

## 2. Materials and Methods

This scoping review was conducted and reported in accordance with the Preferred Reporting Items for Systematic Reviews and Meta-Analyses Extension for Scoping Reviews (PRISMA-ScR) [10,11] (Table S1). Due to its scoping nature, the study protocol did not qualify for registration in the PROSPERO database, and it has not been registered [12]. In line with the objectives of a scoping review, no formal critical appraisal of study quality was conducted, consistent with methodological guidance indicating that scoping reviews are intended to map the extent and nature of the available literature rather than to formally assess the risk of bias or strength of evidence.

### 2.1. Eligibility Criteria

The eligibility criteria included: original research articles; articles involving informal caregivers; articles including caregivers of adult patients with melanoma or sarcoma; articles that assessed the quality of life and/or psychological well-being of caregivers using qualitative or quantitative methods, and studies published in English.

Studies were excluded if they did not specifically address the quality of life or psychological impact experienced by caregivers; they focused only on caregivers of patients with other cancer types; they were not original research (e.g., reviews, editorials, or conference abstracts), or they were published in languages other than English.

### 2.2. Information Sources and Search Strategy

The literature search for this scoping review was conducted from database inception to 21 January 2025. The following electronic databases were searched: PubMed, Embase, and PsycINFO^®^ (via Ovid). All articles published up to the date of the search were considered eligible.

Search terms were developed to capture studies involving caregivers of patients with melanoma or sarcoma and included combinations of disease-related terms (“melanoma”, “sarcoma”) and caregiver-related terms (“caregiver”, “family caregiver”, “informal caregiver”, “spouse”). A comprehensive search strategy was first developed for PubMed and then translated and adapted for Embase and PsycINFO (see Supplementary File Table S2 for the complete search strategy).

The full Boolean search strategy used for PubMed was as follows:

((sarcoma[Title/Abstract]) AND (caregiver[Title/Abstract])) OR ((melanoma[Title/Abstract]) AND (caregiver[Title/Abstract])).

((sarcoma[Title/Abstract]) AND (spouse[Title/Abstract])) OR ((melanoma[Title/Abstract]) AND (spouse[Title/Abstract])).

((sarcoma[Title/Abstract]) AND (informal caregiver[Title/Abstract])) OR ((melanoma[Title/Abstract]) AND (informal caregiver[Title/Abstract])).

((sarcoma[Title/Abstract]) AND (family caregiver[Title/Abstract])) OR ((melanoma[Title/Abstract]) AND (family caregiver[Title/Abstract])).

### 2.3. Study Selection

A total of 325 records were identified across the three databases. All the records retrieved were imported into Rayyan, a web-based platform designed to support systematic and scoping reviews by facilitating blinded screening and duplicate management.

Duplicate records were automatically identified and removed prior to screening. Three reviewers independently screened titles and abstracts in a blinded manner according to the predefined eligibility criteria. Discrepancies were resolved through discussion until consensus was reached. The full texts of potentially eligible studies were subsequently retrieved and independently assessed by the same reviewers using the same consensus-based approach (see Supplementary File Table S3 for the list of articles excluded after full-text review).

**Figure 1:**
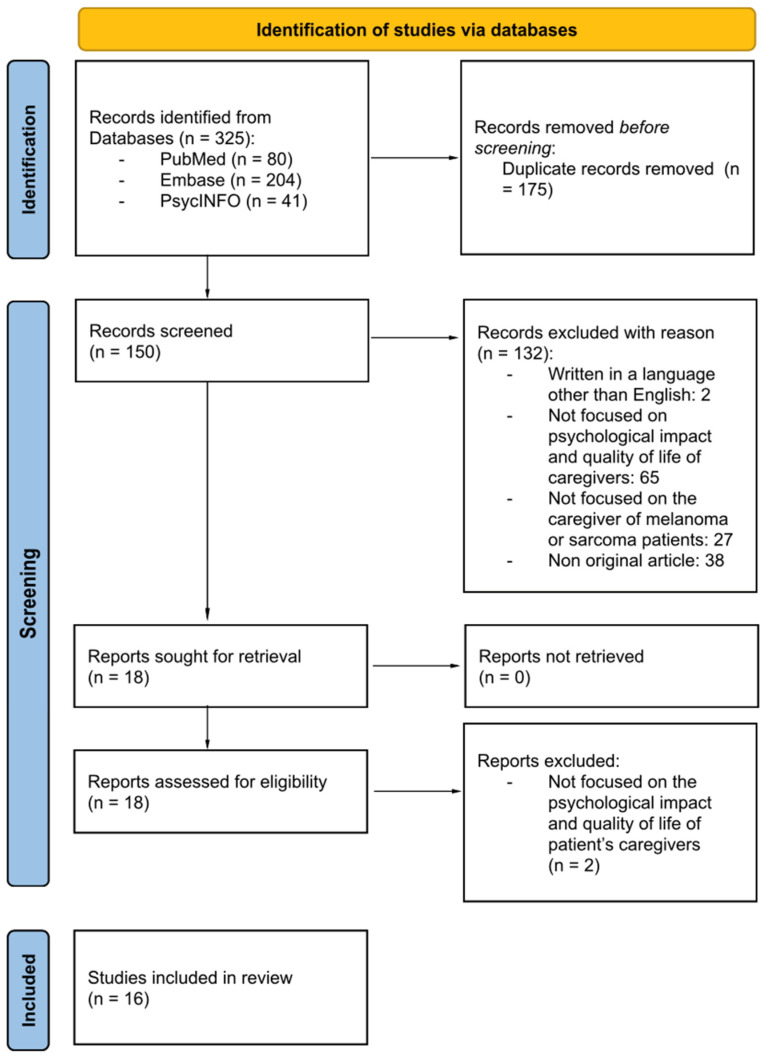
PRISMA screening protocol.

Following full-text screening, 16 studies were included in the final scoping review. Reference lists of included articles were also screened to identify additional relevant studies. The study selection process is summarized in the PRISMA flow diagram (Figure 1).

### 2.4. Data Charting Process and Data Items

Data charting was performed using a predefined extraction form developed specifically for this scoping review.

Data were charted independently by two reviewers, with discrepancies resolved through discussion and, when necessary, consultation with a third reviewer.

The following variables were extracted from each included study (Table S4):

- Study details
  - Authors
  - Title of article
  - Type of publication
  - Year of publication
  - Type of study
- Sample characteristics
  - Sample size
  - Caregiver age
  - Caregiver gender
  - Relationship with the patient
  - Country of study
  - Type of cancer
  - Stage of cancer
  - Educational level (when available)
- Measures
  - Type of assessment
  - Quality of life instruments used for caregivers (when applicable)
  - Quality of life dimensions assessed
  - Psychological assessment instruments used for caregivers (when applicable)
  - Psychological dimensions assessed
- Major findings

### 2.5. Data Synthesis and Analysis

Given the heterogeneity of study designs and outcome measures, findings were synthesized using a descriptive and narrative approach, consistent with scoping review methodology. Extracted data were grouped thematically according to the primary domains assessed, namely quality of life and psychological dimensions of caregiving. The synthesis aimed to map the tools and methods used to assess caregiver well-being and to identify caregiving aspects most consistently associated with increased burden or distress.

## 3. Results

### 3.1. General Characteristics of the Included Studies

The review includes 16 studies published between 2007 and 2024 and conducted on a total of 3464 caregivers. Of these caregivers, 211 were caring for patients with melanoma or sarcoma. While most of the research focuses on melanoma (*n* = 14), only two studies address sarcoma, highlighting a notable gap in the literature on this population.

Importantly, all outcomes reported in this review are derived exclusively from questionnaires, measures, or qualitative interviews completed by caregivers; data collected directly from patients were not considered.

The predominant methodology of the studies was qualitative (9/16), followed by quantitative (5/16), with two studies employing a mixed approach. This heterogeneity reflects the complexity of the issue under study but limits the direct comparability of the results.

The majority of the included caregivers are female (women-to-men ratio of 2:1), with an average age of 50, which is consistent with reports in the general literature on cancer caregiving. In most cases, they are spouses or partners (approximately 54 per cent), followed by children and parents [3.5–19]. The detailed baseline characteristics of the included studies are presented in Table 1 (see Supplementary File Table S5 for more details).

### 3.2. Impact on Quality of Life (QoL)

Quality of life is considered here as an overarching conceptual framework that encompasses the multidimensional impact of caregiving on the daily lives and functioning of caregivers, regardless of whether this impact is assessed using validated quality-of-life instruments or measures of role-related burden and life organization.

All the studies analyzed show that the quality of life of caregivers is significantly compromised, albeit to varying degrees depending on the clinical setting and survey methodology. The main areas of convergence concern the work and economic spheres, which are affected in most studies and often result in a reduced number of working hours, taking leaves of absence, or leaving employment to focus on caregiving [13,19,21]. Financial strain from failing to pay for costs like parking, food, medications, and patient rehabilitation is frequently reported, with caregivers of children or dependents particularly impacted [14,24].

The impact on social and family relationships, leading to social isolation, decreased engagement in recreational activities, and disruption of family roles, is also consistently reported [13,20,23]. Caregivers often need to reorganize their daily routines, balancing caregiving with personal and family responsibilities, especially when patients experience prolonged or unpredictable illness trajectories [9,24]. Time management and daily life are significantly altered, with caregivers dedicating long hours to patient care. For instance, Yabroff et al. [26] reported an average of 8.3 h per day for 13.7 months devoted to caregiving, which also had repercussions on work and personal life.

However, differences emerge in relation to the assessment tools used and the type of disease. Studies employing standardized instruments such as the Family Dermatology Life Quality Index (FDLQI) or the European Organization for Research and Treatment of Cancer Quality of Life Core 30 (EORTC QLQ-C30) tend to reveal a moderate yet consistent impact across various life domains [16,23], including time spent on care, daily activities, social relationships, work and overall physical and psychological well-being.

On the other hand, qualitative research highlights a subjectively greater perceived severity, characterized by descriptions of “emotional exhaustion,” “forced reorganization of life,” pervasive uncertainty, and chronic stress associated with prognostic unpredictability [13,19,22].

Several studies have also identified a recurring divergence between patients’ and caregivers’ perceptions of health-related quality of life (HRQoL), with patients focusing primarily on clinical outcomes and caregivers emphasizing losses in emotional, social, and functional domains [18]. For example, maintaining independence, managing adverse events, and alleviating patient pain were identified by caregivers as central to HRQoL, in addition to their own emotional well-being [18].

### 3.3. Psychological Dimensions

The psychological dimensions are considered to be a broad conceptual domain that encompasses the emotional and cognitive experiences of caregivers in relation to cancer care. These experiences are described using both standardized quantitative tools and qualitative evidence.

These dimensions are a central, cross-cutting theme in all studies. Most research on this topic indicates that caregivers experience high levels of distress, anxiety, and depression [13,23,24], often related to disease severity, prognostic uncertainty, and the unpredictability of treatment outcomes [19,22]. A widespread sense of helplessness, chronic stress, and fear of the future are also reported, particularly among caregivers of patients undergoing immunotherapy or those with advanced melanoma, where clinical progression is less predictable [16,20].

In addition, caregivers tend to deny or postpone requests for psychological support in order to give priority to the patient’s needs [14,16]. Qualitative studies further highlight experiences of emotional exhaustion, pervasive worry, and a forced reorganization of life due to the emotional burden of caregiving [13,14]. Sleep disturbances and fatigue are also commonly reported [9].

The differences emerge mainly between sexes, with female caregivers reporting higher levels of distress and sleep disorders [9], whereas men tend to lean towards social withdrawal.

In addition, protective factors such as spirituality [27] and perceived self-efficacy [9] appear to mitigate psychological distress by serving as buffers between caregiving burden and subjective well-being. Moreover, access to clear information and effective communication with healthcare teams is critical: a lack of adequate information increases anxiety and uncertainty, while well-informed caregivers report lower distress and a greater sense of control over the caregiving process [16,17].

Several studies also indicate that the psychological impact of caregiving is closely intertwined with other life domains, such as work, social relationships, and daily activities. The emotional burden is amplified by financial pressure, disruption of family roles, and the need to reorganize daily life around caregiving responsibilities [14,20,21]. Importantly, discrepancies are often observed between caregivers’ and patients’ perceptions of psychological well-being, with caregivers emphasizing stress, emotional exhaustion, and social isolation, whereas patients focus primarily on clinical outcomes and symptom management [18].

Overall, psychological distress emerges as a central, multidimensional, and persistent aspect of caregiving, shaped by individual factors (sex, coping strategies), relational dynamics, and healthcare system interactions.

Table 2 presents the main findings of the included studies on quality of life and psychological dimensions in caregivers of patients with melanoma and/or sarcoma (see Supplementary File Table S6 for more details).

### 3.4. Instruments and Methods Used to Evaluate Caregiver Quality of Life and Psychological Distress

Across the included studies, a variety of instruments and assessment methods were employed to evaluate caregiver well-being, reflecting both the multidimensional nature of caregiving and the heterogeneity of the literature. Quality of life and caregiver burden were most commonly assessed using standardized tools such as the Family Dermatology Life Quality Index (FDLQI) [23], the Caregiver Reaction Assessment (CRA) [9] and the Work Productivity and Activity Impairment questionnaire for caregivers (WPAI/CG) [16], as well as through qualitative and semi-structured interviews focusing on daily activities, social and family roles, financial impact, and treatment-related responsibilities [14,16,19,20,25]. Psychological outcomes and mood, including anxiety, depression, stress, emotional distress, and coping strategies, were measured using instruments such as the Depression, Anxiety and Stress Scale (DASS-21) [13], the Center for Epidemiologic Studies Depression Scale (CES-D) [9], the Profile of Mood States—Short Form (POMS-SF) [24], and the General Self-Efficacy Scale (GSES) [9], alongside qualitative explorations of hope, uncertainty and perceived control [15,20,22]. Some studies, such as those by Thompson et al. [16], used patient-reported measures, such as the EORTC QLQ-C30, with caregivers, which represent an indirect approach in the absence of validated caregiver-specific instruments. Overall, this variety of tools highlights the complexity of assessing caregiver well-being and underscores the need for standardized, comprehensive instruments that capture both the practical and emotional dimensions of caregiving. Table 3 summarizes all the instruments used in the articles (see Supplementary File Table S7 for more details).

### 3.5. Methodological Differences and Critical Implications

A comparison of the included studies reveals significant methodological differences that should be carefully considered when interpreting the findings. The psychometric instruments used to assess caregiver burden, psychological distress, and quality of life vary considerably between the studies, limiting the possibility of direct comparisons and meta-analytical synthesis.

Qualitative studies provide a more in-depth and nuanced understanding of caregivers’ subjective experiences, emotional responses, and coping strategies, albeit with limited generalizability due to small and context-specific samples. In contrast, quantitative studies offer more standardized and comparable data, though they may fail to fully capture the complexity and dynamic nature of the caregiving experience, particularly with rare and aggressive cancers. Moreover, only a few studies analyze the evolution of caregiver well-being over time [9], making it difficult to understand how psychological distress and quality-of-life impairment progress through the various stages of the disease trajectory and treatment pathways.

Despite these methodological limitations, there is substantial convergence between studies in emphasizing that caregiving for patients with melanoma or sarcoma has a major multidimensional impact—encompassing psychological, relational, occupational, and economic domains—which underscores the need for structured, integrated, and caregiver-centered support interventions.

## 4. Discussion

The aim of this review was to synthesize the available evidence regarding the impact of melanoma and sarcoma on caregivers’ quality of life and psychological well-being. By analyzing 16 studies published between 2007 and 2024 and involving 3464 caregivers, the findings suggest that caregiving in this cancer setting, as described primarily in studies involving mixed cancer caregiver populations, is associated with a substantial and multidimensional burden. Although often used interchangeably in the literature, quality of life, psychological distress, caregiver burden, and unmet needs represent distinct but overlapping conceptual domains. In the studies reviewed, quality of life is predominantly associated with disruptions in daily functioning, employment, and social roles. Psychological distress is characterized by emotional and cognitive symptoms, including anxiety, depression, and chronic stress. Caregiver burden reflects the intensity and perceived weight of caregiving responsibilities, while needs relate to gaps in information, psychological support, and healthcare communication. Importantly, although melanoma is relatively well represented in the literature, the limited number of studies focusing on sarcoma highlights a critical knowledge gap, due to the rarity, heterogeneity, and clinical aggressiveness of this disease.

### 4.1. Theoretical Framework and Significance of the Results

The psychological outcomes observed across studies can be interpreted within Lazarus and Folkman’s transactional model of stress and coping [25], which conceptualizes stress as the result of a dynamic interaction between environmental demands and individual coping resources. Caregivers of patients with melanoma and/or sarcoma, as described in the included literature—often based on mixed cancer samples—are exposed to prolonged and unpredictable stressors, including uncertainty regarding prognosis, fluctuating treatment responses—particularly in immunotherapy—and emotional involvement in the patient’s illness trajectory.

The analysis of the studies revealed that high levels of distress, anxiety, and depressive symptoms were consistently reported [13,23,24], especially when disease severity and unpredictability were pronounced [20]. Within the transactional framework, these outcomes suggest that caregiving demands often exceed perceived coping resources, particularly when caregivers lack adequate informational, social, or psychological support. This imbalance may be exacerbated in rare cancers such as sarcoma; however, direct evidence remains limited due to the small number of sarcoma-specific studies.

The stress process model developed by Pearlin et al. [28] offers an additional explanatory lens by framing caregiving as a cumulative process in which primary stressors (e.g., care intensity, symptom management) interact with secondary stressors, including role strain, financial difficulties, and social isolation. The convergence of psychological, occupational, and economic impacts reported across the studies reviewed strongly supports this model and underscores the chronic and progressive nature of caregiver burden.

In light of the transactional model of stress and coping [29] and the caregiver stress model [28], the data suggest that cancer caregiving should be understood as a dynamic and multifaceted process rather than a purely practical task. It represents a complex psychological experience shaped by ongoing cognitive appraisal and adaptive efforts. Perceived resource availability, the meaning attributed to the disease, and access to social support play a central role in determining psychological outcomes. When caregivers experience a mismatch between caregiving demands and available resources, distress levels increase, while limited resources, insufficient information, and barriers to support services further intensify perceived burden and undermine coping capacity.

### 4.2. Sex Differences and Vulnerability Factors

In the studies analyzed, women constituted the majority of caregivers, with an average age of around 50 years and a women-to-men ratio of approximately 2:1. This distribution is consistent with European data on informal caregiving. It reflects ongoing sex disparities in the allocation of care responsibilities. However, sex differences extend beyond prevalence to encompass qualitative differences in psychological responses and coping patterns.

Female caregivers consistently report higher levels of emotional distress, anxiety, and sleep disturbances [5], whereas male caregivers tend to exhibit social withdrawal and reduced emotional expression. These findings are consistent with broader cancer caregiving literature and suggest that sex acts as a moderator of caregiving outcomes, probably influenced by social role expectations, differential access to support, and culturally shaped coping strategies [30,31,32]. From a clinical perspective, this highlights the importance of moving beyond a “one-size-fits-all” approach and adopting sex-sensitive screening and intervention strategies.

### 4.3. Quality of Life Impairment and Occupational Consequences

Across all studies, caregivers’ quality of life was significantly compromised, although most of the evidence derives from mixed cancer caregiver populations rather than melanoma or sarcoma-specific samples. The occupational and economic domains are the most consistently affected, with reductions in working hours, periods of absence, and job abandonment frequently reported [9,16,18]. These findings are consistent with international evidence on cancer caregiving and reinforce the concept of ‘financial toxicity’ as an issue that affects not only patients, but also their informal care networks [33,34].

Qualitative studies tend to describe a particularly severe subjective impact, characterized by emotional exhaustion, identity disruption, and forced life reorganization. In contrast, quantitative studies using standardized instruments such as the FDLQI or EORTC QLQ-C30 revealed a moderate but pervasive impairment across multiple life domains. This discrepancy underscores the complementary value of qualitative and quantitative approaches while it also highlights the limitations of existing psychometric tools in capturing the full complexity of caregiver experiences, especially in rare cancer settings.

### 4.4. Psychological Distress, Uncertainty, and Communication

Psychological distress emerged as a central, cross-cutting theme across all included studies. Caregivers frequently reported feelings of helplessness, anticipatory anxiety, and fear of disease progression, particularly in situations characterized by clinical uncertainty, such as immunotherapy with unpredictable outcomes. Notably, several studies indicate that caregivers often postpone or deny their own psychological needs in order to give priority to the patient’s care [14,16], potentially increasing long-term vulnerability and risk of burnout.

Inadequate communication with healthcare professionals is a recurrent, modifiable determinant of distress identified across many of the studies considered. A lack of clear, timely, and comprehensible information amplifies uncertainty and undermines caregivers’ perceived ability to cope [16,17]. Conversely, effective communication, characterized by continuity, transparency, and recognition of the caregiver’s role, appears to promote a sense of control and psychological adaptation. These findings are consistent with psycho-oncological models advocating participatory and family-centered communication strategies [35]. Scientific literature suggests that caregivers prefer and benefit from communication that acknowledges the needs of both patients and family members and involves family members in communication and decision-making [36].

The studies reviewed also suggest that the nature and intensity of caregiver distress vary between disease stages. Caregiving during early or potentially curative stages appears to be characterized by role reorganization and uncertainty, particularly in relation to daily life management and future planning [9,24]. In contrast, advanced disease and immunotherapy settings are associated with heightened anticipatory anxiety, fear of progression, and emotional exhaustion, reflecting greater prognostic unpredictability [16,19,20,22].

### 4.5. Methodological Diversity and Gaps in Assessment

A recurring theme in the studies analyzed is the fragmentation of methodological approaches and the limited availability of validated tools for assessing the well-being of caregivers of patients with rare cancers. This variability reflects the limited theoretical consolidation of the field and the small number of studies available. Across studies, a wide range of instruments was used, including standardized tools for quality of life and caregiver burden (FDLQI [23], CRA [9], WPAI-CG [21]), psychometric scales for psychological outcomes (DASS-21 [16], CES-D [9], POMS-SF [27], GSES [9]), and qualitative interviews capturing daily activities, social roles, and financial impact [13,14,15,19,20,22,25]. Some studies also used patient-reported outcome measures designed for patients (e.g., the EORTC QLQ-C30) with caregivers [16], reflecting the lack of validated caregiver-specific tools.

The instruments identified measured a wide variety of constructs and can be grouped into distinct families: quality of life measures (e.g., FDLQI, EORTC QLQ-C30), caregiver burden scales (e.g., CRA, WPAI-CG), psychological distress assessments (e.g., DASS-21, CES-D, POMS-SF), and qualitative interviews capturing unmet needs and supportive care requirements.

The psychometric properties of these tools also varied: some, such as the FDLQI and CRA, have been validated in caregiver populations, whereas others were adapted from patient-focused measures or lacked full reliability and validity assessments, highlighting the need for standardized and caregiver-specific instruments.

This heterogeneity highlights both the complexity of assessing caregiver experiences and the current lack of standardized, culturally sensitive, and disease-specific instruments for melanoma and sarcoma caregivers. The diversity of tools, study designs, and outcome measures limits direct comparability and synthesis. Future research should therefore prioritize the development of comprehensive instruments capable of capturing the practical and emotional dimensions of caregiving, including burden, psychological distress, resilience, coping strategies, and positive adaptation. Longitudinal, multicentric studies using such tools are needed to provide a more nuanced and generalizable understanding of caregiver experiences with rare cancers.

Notably, several domains relevant to caregiver well-being—such as resilience, positive coping strategies, and social functioning—were infrequently assessed, representing a consistent gap across studies.

Differences in instrument use and domains assessed between melanoma and sarcoma caregivers were also observed: while melanoma caregivers were evaluated with a broader range of tools across multiple domains, sarcoma-specific studies were sparse and often limited to generic QoL or distress measures, underscoring the need for disease-specific assessment.

Taken together, these methodological limitations and gaps in both instrument coverage and disease-specific assessment underscore the urgent need for standardized, comprehensive tools and targeted research to better capture the multifaceted experiences of caregivers.

### 4.6. Clinical and Policy Implications

Despite the methodological limitations, the consistency of findings across diverse study designs supports the conclusion that caregivers of patients with melanoma and/or sarcoma may constitute a high-risk population; however, this conclusion is largely based on evidence from mixed cancer caregiver samples and should therefore be interpreted with caution. Clinically, this highlights the need for integrated interventions that combine routine psychological screening, caregiver-focused psychoeducational programs, and accessible digital information tools co-designed with caregivers. The evidence underlines the importance of coordinated psychosocial approaches combining emotional support, skill-based training, and practical assistance. Such interventions should be tailored to the specific stage of the disease and to caregivers’ individual vulnerability profiles, rather than delivered as uniform support measures; nevertheless, disease-specific recommendations for melanoma and sarcoma caregivers remain preliminary due to limited targeted evidence. Promoting participatory communication models among healthcare teams, patients, and caregivers is essential to improving quality of life and reducing distress, particularly by increasing caregiver involvement in care planning.

Furthermore, structured interventions incorporating group support programs, counseling, and digital technologies can effectively support caregivers in the long term. Digital and hybrid interventions may be especially valuable for caregivers of patients with rare cancers, who often experience geographical and organizational barriers to specialized support access.

From a policy perspective, the documented occupational and economic consequences highlight the need for supportive workplace policies and social protection measures that recognize informal caregiving as a critical component of cancer care. It is not only ethically imperative to address the needs of caregivers, but also essential to ensure the sustainability and effectiveness of cancer care systems.

### 4.7. Study Limitations

This review has several limitations that should be acknowledged. Firstly, the relatively limited number of studies included in this review has a significant impact on the robustness and generalizability of the findings. Nevertheless, the final number of included studies is limited, reflecting, however, the strict inclusion criteria and the focused research issue addressed by this review.

Furthermore, only two of these studies focus specifically on caregivers of sarcoma patients, while the remaining literature predominantly addresses caregivers of melanoma patients. This limits the representativeness of the findings for rare cancers. Furthermore, the samples were not fully diverse in terms of sex and ethnic origin. There was a predominance of female caregivers, and the studies were mainly conducted in European or North American countries. This may further restrict the generalizability of the results, particularly in other parts of the world with different cultural norms, healthcare systems, and caregiver roles, as evidence from low- and middle-income countries was scarce and limited to a single study, precluding meaningful cross-country or income-level comparisons.

We also acknowledge a major internal validity consideration: while the review question focuses on caregivers of melanoma and sarcoma patients, the majority of included studies are based on mixed-cancer samples. This means that many of the conclusions drawn may not fully reflect the experiences of melanoma or sarcoma caregivers, particularly those with sarcoma, where only two studies were available. However, a limited number of studies specifically conducted on caregivers of melanoma or sarcoma patients have reported findings that are largely consistent with the results from mixed-cancer samples. This observation lends support to the plausibility that the patterns of caregiver burden and psychological impact identified in the broader literature are also relevant to our target populations. Nevertheless, this emphasizes the urgent need for further research expressly focused on melanoma and, especially, sarcoma caregivers to strengthen disease-specific evidence.

Moreover, the heterogeneity of the study designs, including qualitative, quantitative, and mixed-method approaches, as well as the use of various assessment tools and outcome measures, makes direct comparisons between studies difficult. Variability across instruments, some adapted from broader cancer settings without validation for melanoma or sarcoma caregivers, further limits interpretability and synthesis. Furthermore, the limited availability of instruments specifically validated for use with caregivers in melanoma and sarcoma settings is a significant methodological constraint. Although patient-reported outcome measures such as the EORTC QLQ-C30 can provide useful information when extended to caregivers, they may not fully capture caregiver-specific dimensions of quality of life and burden.

The predominance of qualitative and cross-sectional designs restricts comparability and the ability to draw causal inferences. Furthermore, only a few studies examined caregiver outcomes longitudinally, leaving significant gaps in our understanding of how psychological distress and quality of life deteriorate through the various stages of the disease. These methodological differences complicate the synthesis of results and may contribute to inconsistencies in reported outcomes, underlining the importance of developing standardized, culturally sensitive, and validated assessment tools for future research.

Taken together, these limitations highlight the need for more standardized, longitudinal, and multicenter research involving larger, more diverse samples, paying particular attention to caregivers of patients with rare cancers, such as sarcoma. Such studies represent essential input for evidence-based interventions, healthcare planning, and policy decisions aimed at mitigating caregiver burden and improving their quality of life.

## 5. Conclusions

This scoping review aims to map the existing evidence on the impact of caring for patients with melanoma or sarcoma. It highlights multiple interrelated domains, including quality of life, psychological distress, caregiver burden, and unmet needs. These domains are assessed (e.g., FDLQI, CRA, WPAI/CG, DASS-21, CES-D, POMS-SF, GSES, and EORTC QLQ-C30). However, the available evidence is predominantly derived from studies involving mixed cancer caregiver populations. Across the literature reviewed, these domains do not operate independently but interact over time, contributing to an overall caregiving burden that may intensify along the disease trajectory. The evidence that has been mapped suggests an increased emotional vulnerability and a potential risk of chronic distress, particularly in settings characterized by clinical uncertainty and prolonged treatment pathways.

The following key gaps have been identified through this mapping exercise: firstly, there is a scarcity of melanoma- and sarcoma-specific caregiver studies, especially for sarcoma; secondly, there is a preponderance of cross-sectional designs; and thirdly, there is limited availability of validated caregiver-specific instruments for rare cancers. Consequently, research priorities should include longitudinal studies to analyze the evolution of distress and coping strategies over time, the recruitment of larger, disease-specific samples, and the adoption of mixed theoretical and methodological approaches integrating quantitative outcomes with qualitative insights.

Future studies should also adopt mixed theoretical and methodological approaches that integrate quantitative outcomes with qualitative insights, thereby capturing both the subjective and objective dimensions of the caregiving experience. In this perspective, to draw more robust and disease-specific conclusions, it is essential to design studies expressly focused on caregivers of patients with rare cancers. In particular, further research focusing on caregivers of patients with sarcoma is urgently needed, as only two sarcoma-specific studies are currently available in the literature.

We also acknowledge that the search results included in this review are now over a year old, and updating the literature is an important objective for future work to ensure the timeliness and completeness of the evidence.

In conclusion, this scoping review provides a structured overview of the psychological and social challenges reported by caregivers of patients with melanoma and/or sarcoma, while emphasizing the need to interpret these findings in light of current evidence gaps. By mapping existing domains, tools, and methodological approaches, the review highlights priorities for future research. It is argued that this will support the development of more targeted, evidence-based caregiver support strategies within psycho-oncology, particularly in rare and complex cancer settings.

## Figures and Tables

**Table 1:** Baseline characteristics of the included studies: study details and sample characteristics.

Author	Title	Year	Study Design	Number of Patients (Caregivers)	Relationship	Type of Cancer
Mancini J. [13]	Quality of life in a heterogeneous sample of caregivers of cancer patients: An in-depth interview study.	2011	Qualitative Research	77 (17 melanoma)	2 parent, 12 spouse, 2 child, 1 friend	breast cancer, melanoma, paediatric haematology
Weaver R. [14]	The unmet needs of carers of patients diagnosed with sarcoma: A qualitative study.	2021	Qualitative Research	33	15 mother, 4 father, 11 spouse, 2 brother, 1 daughter	sarcoma
Fox J.A. [15]	Palliative care in the context of immune and targeted therapies: A qualitative study of bereaved carers’ experiences in metastatic melanoma.	2020	Qualitative Research	20	16 partner, 2 sibling, 2 child	metastatic melanoma
Thompson J.R. [16]	Supportive care needs in Australian melanoma patients and caregivers: results from a quantitative cross-sectional survey.	2023	Quantitative Cross-sectional Survey	37	29 partner, 8 immediate family member	melanoma
Marshall-McKenna R. [17]	A multinational investigation of healthcare needs, preferences, and expectations in supportive cancer care: Co-creating the LifeChamps digital platform.	2022	Descriptive, Cross-sectional, Multi-method study.	23	10 daughter, 8 spouse/partner, 3 other, 1 sister in law, 1 son	breast cancer, prostate cancer, melanoma
Makady A. [18]	Social media as a tool for assessing patient perspectives on quality of life in metastatic melanoma: A feasibility study.	2018	Survey	17	-	melanoma
Johansen S. [9]	The effect of cancer patients’ and their family caregivers’ physical and emotional symptoms on caregiver burden.	2018	Cross-sectional quantitative research	281 (42 head neck and skin)	227 spouse/partner, 45 family member, 9 other family member	breast, prostate, melanoma, myelomatose, lymphoma, head-neck cancers
Milne D. [19]	Exploring the experiences of people treated with immunotherapies for advanced melanoma and those caring for them: ‘Real-world’ data.	2020	Qualitative Research, Cross-sectional	9	-	melanoma
Shilling V. [20]	The pervasive nature of uncertainty—A qualitative study of patients with advanced cancer and their informal caregivers.	2017	Qualitative Research	8	spouse/partner	ovarian, melanoma, lung cancer
Aguiar-Ibanez R. [21]	Impact of recurrence on employment, finances, and productivity for early-stage cancer patients and caregivers: US survey	2024	Cross-sectional, Non-interventional, Online survey	100 (17 melanoma)	48 spouse/significant other, 24 son/daughter, 10 parent, 9 friend/neighbour, 9 sibling	bladder, gastric, head and neck, non–small cell lung, renal cell, triple-negative breast cancers, melanoma
Boulanger M.C. [22]	Patient and caregiver experience with the hope and prognostic uncertainty of immunotherapy: A qualitative study	2024	Qualitative Research	10 (7 melanoma)	8 Spouse, 1 sibling, 1 other family member	melanoma, NSCLC
Papanikolaou E.S. [23]	Quality of life in caregivers of melanoma patients	2022	-	120	50 son/daughter, 23 partner, 27 brother/sister, 20 other	melanoma
Muliira J.K. [24]	Roles of family caregivers and perceived burden when caring for hospitalized adult cancer patients: Perspective from a low-income country	2018	Cross sectional, Descriptive design	168	46 spouse, 122 not spouse	kaposi’s sarcoma, prostate carcinoma, leukemia, pancreatic cancer, esophageal cancer, bone cancer, seminoma, hepatocarcinoma, colorectal cancer, breast cancer
Tan J.D. [25]	A qualitative assessment of psychosocial impact, coping and adjustment in high-risk melanoma patients and caregivers	2014	Qualitative Research	14	8 partner, 1 parent, 1 child, 2 friend, 2 other	melanoma
Yabroff K.R. [26]	Time costs associated with informal caregiving for cancer survivors	2009	Qualitative research	688 (73 bladder, skin and uterine)	451 spouse/partner, 111 child/child-in-law, 29 parent, 57 sibling, 26 friend, 14 other	bladder, breast, colorectal, kidney, lung, melanoma of the skin, ovarian, prostate, or uterine cancer, non-Hodgkins lymphoma (NHL)
Kim Y. [27]	Psychological distress of female cancer caregivers: Effects of type of cancer and caregivers’ spirituality	2007	Qualitative research	1635 (7 melanoma)	28 mother, 60 sister, 110 daughter, 21 friend, 8 daughter-in-law, 6 other in-law, 7 partner, 12 other (just female)	breast, kidney, lung, non-Hodgkin’s lymphoma, melanoma, ovarian cancer.

**Table 2:** Main findings of the included studies on quality of life and psychological dimensions in caregivers of patients with melanoma and/or sarcoma.

Author	Assessment Type	Health-Related Quality of Life (HRQoL) Measures	HRQoL Dimensions	Psychological Measures	Psychological Dimensions	Major Findings
Mancini J. [13]	Qualitative	Semi-structured Interviews	Leisure and daily activities; occupation and financial issues; physical well-being; relationship with healthcare professionals; relationship with family and friends; patient–caregiver relationship; relationships with institutional caregivers	Semi-structured Interviews	Psychological well-being: anxiety, fear, stress, sadness, depression, insecurity, and perceived injustice or unfairness; relationship with the patient: intimacy, communication, and emotional dynamics	In line with previous literature, the study found that caregiving was primarily associated with a significant psychological burden, even in cases involving patients with favorable prognoses, while serious physical consequences were rare. The findings highlight the need for a standardized assessment of caregivers’ quality of life, supplemented by specific modules for cancer and relationships.
Weaver R. [14]	Qualitative	Semi-structured Interviews	Support with Medical Aspects of Caregiving: Medical tasks and care-related support; Need for Information about the Patient: Access to patient-related information; Financial Impact: Economic burden of caregiving	Semi-structured Interviews	Psychological Support for Caregivers and Family: Emotional and psychological support needs of caregivers and family members	Caregivers of sarcoma patients report that the patients’ needs, which also affect them, are not being met in several areas: medical, informational, psychosocial and financial. Many caregivers have experienced psychological distress but have struggled to seek support, prioritising the needs of their patients, which has had a negative impact on their quality of life and ability to provide care. It is therefore necessary to develop support programmes tailored to caregivers, including financial assistance and support groups.
Fox J.A. [15]	Qualitative	-	-	Semi-structured Interviews (Grounded Theory)	Psychological dimensions: hope-related expectations, treatment continuation despite uncertain benefit, unmet informational needs, delayed integration of palliative care, and lack of end-of-life planning	The investigation reveals that care providers encounter a variety of difficulties, especially when it comes to acquiring clear infomrations from medical practitioners, for example during the transition to end-of-life care. Several factors contribute to caregivers’ feelings of overwhelm and stress, including understanding complex prognostic data, navigating the debate between palliative and continuing care, and a lack of preparation for the end of life of the person they are caring for. These findings highlight the ongoing need for information, psychosocial support and preparation, and emphasise the importance of educational initiatives and improved communication between doctors, patients and caregivers to alleviate the burden on caregivers.
Thompson J.R. [16]	Mixed-Methods	European Organisation for Research and Treatment of Cancer Quality of Life Questionnaire (EORTC QLQ-C30); Supportive Care Needs Survey Partners&Caregiver (SCNS-P&C)	Caregiver quality of life: healthcare-related needs, work and social functioning, and global health	Depression, Anxiety and Stress Scale (DASS-21)	Psychological distress: anxiety, depression, stress, and distress related to unmet information needs	The study revealed that Australian melanoma patients and their caregivers have significant unmet psychological and emotional needs. The findings highlight the need for a standardized assessment of caregivers’ quality of life, supplemented by specific modules on cancer and relationships. Focusing on these aspects would help facilitate the resolution of the issues identified.
Marshall-McKenna R. [17]	Mixed-Methods	Online Survey	Pervasive Uncertainty: Widespread and ongoing uncertainty across care trajectories	Online Survey	Psychological Needs: Need for psychological support; Relationship with Institutions and Information Needs: Interaction with institutions and access to information.	The analysis conducted by the authors shows that cancer survivors and their family caregivers report dissatisfaction with the lack of clear information and ongoing support throughout the cancer journey. Given these gaps, follow-up services and targeted psychological support are essential to address these shortcomings.
Makady A. [18]	Quantitative	Qualitative Interviews	Family and Social Relationships; Emotional Burden: Fear and concerns.	/	/	This study explored the feasibility of using social media to assess the perspectives of patients and their caregivers on health-related quality of life (HRQoL). The research found that caregivers prioritized the ability to cope with manageable adverse events, to be capable, and to be free from pain, while patients prioritized family, emphasizing the importance of leading a normal life and enjoying life. Some also emphasized the importance of not neglecting career-related aspects.
Johansen S. [9]	Quantitative	Caregiver Reaction Assessment (CRA); Medical Outcomes Study Social Support Survey (MOS-SSS); General Self-Efficacy Scale (GSES); General Sleep Disturbance Scale (GSDS)	Fatigue; Sleep Disturbance; Symptom Distress	Caregiver Reaction Assessment (CRA); Lee Fatigue Scale (LFS); Center for Epidemiologic Studies Depression Scale (CES-D)	Psychological and social outcomes: fatigue, depression, sense of self-efficacy, stress and emotional distress, perceived social support	The research found that a significant burden on caregivers is associated with depression, fatigue, sleep disturbances, low self-esteem and limited social support in patients. The survey found that female patients and caregivers experienced a greater burden than men. These findings emphasise the importance of including caregivers in the cancer care pathway.
Milne D. [19]	Qualitative	Qualitative Interviews	Economic and physical burden: financial toxicity and fatigue	Qualitative Interviews	Treatment-related uncertainty and associated anxiety	This research focuses on patients receiving immunotherapy for stage IV melanoma and their caregivers, who report experiencing a reduced quality of life due to treatment-related toxicities, stress, financial hardship and fatigue. Key challenges include uncertainty and an increased burden of caregiving responsibilities and side effects. The findings highlight the need for comprehensive preparation, clear information and rapid access to knowledgeable healthcare professionals to support patients and their caregivers.
Shilling V. [20]	Qualitative	Qualitative Interviews	Job and financial implications: concerns related to employment, loss of earnings, and perceived financial position Implications for the future: changes in outlook, realigning priorities, life on hold, opportunities lost, and inability to plan for the future	Qualitative Interviews	Managing uncertainty: control, preservation of or return to normality, hope, and mindset Relationships and communication: patient–caregiver relationship and communication, prevalence of cancer-related conversations, and family dynamics	Patients and caregivers faced significant uncertainty about the future, leading to loss of control over work, finances, family, and retirement. Coping focused on maintaining “normality,” with family well-being prioritized by patients and caregivers feeling their lives “on hold.” Impact varied by age and closeness to the patient, highlighting the need for open communication and targeted support.
Aguiar-Ibanez R. [21]	Qualitative	Online Survey; Work Productivity and Activity Impairment: Caregiver (WPAI:CG)	Work productivity and activity impairment	Online Survey	/	Cancer recurrence has a substantial negative impact on work productivity, employment stability, and financial well-being for both patients and caregivers. These challenges contribute to increased stress, limit the ability to participate in daily activities, and exacerbate the overall burden of caregiving, highlighting the need for targeted support and interventions to mitigate these effects.
Boulanger M.C. [22]	Qualitative	Qualitative Interviews	Disruption of family and occupational roles	Qualitative Interviews	Hope and prognostic uncertainty; overwhelming disappointment among patients without long-term response; conflicting preferences for receiving prognostic information; chronic stress related to ongoing prognostic uncertainty and constant vigilance; perceived lack of control over the course of treatment and the patient’s prognosis	Optimistic expectations were often influenced by oncology teams, but uncertainty and unpredictable long-term treatment responses caused emotional distress and disappointment. Patients and caregivers had differing preferences for prognostic information, underscoring the need for personalized communication strategies.
Papanikolaou E.S. [23]	Quantitative	Family Dermatology Life Quality Index (FDLQI)	Time dedicated to caregiving; impact on work or studies; social activities and leisure time; personal relationships; daily activities; financial aspects; sleep and rest; overall impact on quality of life	Family Dermatology Life Quality Index (FDLQI)	Emotional and psychological well-being; self-perception in the caregiver role	Emotional distress was identified as the primary contributor to caregiver burden on the FDLQI. Burden was higher for son/daughter caregivers and increased with patient age and time since diagnosis, whereas caregiver sex, age, and educational level showed no significant effect.
Muliira J.K. [24]	Quantitative	/	/	Caregiver Burden Scale (CBS)	Psychological dimensions: emotional and psychological well-being; self-perceived role and identity as a caregiver	Caregivers in low-income settings experience high, multidimensional burden, including physical, emotional, and social impacts. Key interventions to alleviate this burden include emotional support, training, and practical assistance such as dedicated nursing care.
Tan J.D. [25]	Qualitative	Semi-structured Phone Interviews	Treatment impact: disruption of daily life, including occupational, social, and family roles; financial impact; caregiver role devaluation	Semi-structured Phone Interviews	Emotional distress; feelings of isolation, helplessness, and lack of support from healthcare systems; coping strategies, including adaptive (support, helpful thinking, meaning-making) and maladaptive (avoidance, suppression)	Patients and caregivers reported significant emotional distress across all disease phases, including shock, anxiety, fear, sadness, and frustration. Caregivers assumed new roles during treatment, leading to feelings of inadequacy, guilt, and being overwhelmed, with some experiencing devaluation of their own struggles. Coping strategies varied by disease phase. Findings highlight the need for routine psychological screening, enhanced communication, and targeted supportive care for both patients and caregivers.
Yabroff K.R. [26]	Quantitative	Survey	Time costs related to caregiving; financial impact	Survey	Emotional support and instrumental support (providing practical help and assistance)	Caregiving for cancer patients imposes substantial time demands, averaging 8.3 h per day over 13.7 months, with the highest burden for patients with distant disease. About half of caregivers provided emotional, instrumental, tangible, or medical support, highlighting the significant time-related burden and costs in the first two years after diagnosis.
Kim Y. [27]	Qualitative	FACIT-Sp	Spiritual Well-Being	Profile od Mood States (POMS-SF); Pearlin Stress Scale;	Psychological dimensions: caregiving stress; caregiver spirituality; caregiver psychological distress	Caregivers of survivors with nongender-specific cancers experienced higher psychological distress than those caring for gender-specific cancers. Increased caregiving stress and lower spirituality were linked to greater distress, whereas higher spirituality mitigated stress effects, emphasizing the influence of cancer type and personal spiritual resources on caregiver adjustment.

**Table 3:** Instruments and Methods Used to Evaluate Caregiver Quality of Life and Psychological Distress across the articles.

Assessment Tools	Construct Assessed	QoL or Psychological Domains	Target Population	Psychometric Informations in Caregiver Populations	Studies Using the Instrument Included in the Revision Paper	Population on Which the Instrument Was Administered
European Organisation for Research and Treatment of Cancer Quality of Life Questionnaire (EORTC QLQ-C30)	Helath Related Quality of Life	QoL	Adult cancer patients	Not validated on caregivers population	Thompson J.R. [16]	Caregivers
Supportive Care Needs Survey for Partners and Caregivers (SCNS-P&C)	Unmet supportive care needs of partners and caregivers	Both	Partners and caregivers of cancer survivors	Validated on caregivers population	Thompson J.R. [16]	Caregivers
Depression, Anxiety and Stress Scale—21 Items (DASS-21)	Depression, anxiety, and stress	Psychological Domains	General/Clinical population	Not validated on caregivers population	Thompson J.R. [16]	Caregivers
Caregiver Reaction Assessment (CRA)	Caregiving burden	Psychological Domains	General/Clinical population	Validated on caregivers population	Johansen S. [9]	Caregivers
Profile of Mood States—Short Form (POMS-SF)	Mood	Psychological Domains	General population	Validated on caregivers population	Kim Y. [27]	Caregivers
Family Dermatology Life Quality Index (FDLQI)	Impact of skin diseases on family quality of life	QoL	Family members of dermatology patients	Validated on caregivers population	Papanikolaou E.S. [23]	Caregivers
Medical Outcomes Study Social Support Survey (MOS-SSS)	Perceived social support	Psychological Domains	Patients with chronic illnesses	Not validated on caregivers population	Johansen S. [9]	Caregivers
General Self Efficacy Scale (GSES)	General self-efficacy	Psychological Domains	General population	Not validated on caregivers population	Johansen S. [9]	Caregivers
General Sleep Disturbance Scale (GSDS)	Incidence and nature of sleep disturbance	QoL	General population	Validated on caregivers population	Johansen S. [9]	Caregivers
Lee Fatigue Scale (LFS)	Fatigue severity	QoL	General/Clinical population	Validated on caregivers population	Johansen S. [9]	Caregivers
Center for Epidemiologic Studies Depression Scale (CES-D)	Presence and severity of depressive symptoms	Psychological Domains	General population	Validated on caregivers population	Johansen S. [9]	Caregivers
Caregiver Burden Scale (CBS)	Caregiver burden	Both	Caregiver population	Validated on caregivers population	Muliira J.K. [24]	Caregivers
Pearlin Role Overload Measure (Pearlin ROM)	Stress	Psychological Domains	Caregiver population	Validated on caregivers population	Kim Y. [27]	Caregivers
FACIT-Sp12	Spritual Well-being	QoL	Clinical Population	Validated on caregivers population	Kim Y. [27]	Caregivers
Work Productivity and Activity Impairment: Caregiver (WPAI:CG)	Work and activity impairment	QoL	Patients with health condition	Validated on caregivers population	Aguiar-Ibanez R. [21]	Caregivers

## Data Availability

No new data were created or analyzed in this study.

## Supplementary Materials

The following supporting information can be downloaded at https://www.mdpi.com/article/10.3390/cancers18050809/s1. Table S1: PRISMA-ScR Checklist; Table S2: Full search strings; Table S3: List of excluded articles after full-text reading; Table S4: Data extraction form; Table S5: Baseline characteristics of the included studies: study details and sample characteristics; Table S6: Main findings of the included studies on quality of life and psychological dimensions in caregivers of patients with melanoma and/or sarcoma; Table S7: Instruments and Methods Used to Evaluate Caregiver Quality of Life and Psychological Distress across the articles. References [28,37,38,39,40,41,42,43,44,45,46,47,48,49,50,51,52] are cited in the supplementary materials.

## References

[1] Cassalia F., Cavallin F., Danese A., Del Fiore P., Di Prata C., Rastrelli M., Mocellin S. (2023). Soft tissue sarcoma mimicking melanoma: A systematic review. Cancers.

[2] Wang X., Ma S., Zhu S., Zhu L., Guo W. (2025). Advances in Immunotherapy and Targeted Therapy of Malignant Melanoma. Biomedicines.

[3] Wang M., Gao X., Zhang L. (2025). Recent global patterns in skin cancer incidence, mortality, and prevalence. Chin. Med. J..

[4] Gronchi A., Miah A.B., Dei Tos A.P., Abecassis N., Bajpai J., Bauer S., Biagini R., Bielack S., Blay J.Y., Bolle S. (2021). Soft Tissue and Visceral Sarcomas: ESMO–EURACAN–GENTURIS Clinical Practice Guidelines for Diagnosis, Treatment and Follow-Up. Ann. Oncol..

[5] Amaral T., Ottaviano M., Arance A., Bastholt L., Bhatia S., Berking C., Eigentler T., Garbe C., Grob J.-J., Hauschild A. (2025). Cutaneous Melanoma: ESMO Clinical Practice Guideline for Diagnosis, Treatment and Follow-Up. Ann. Oncol..

[6] Associazione Italiana di Oncologia Medica (AIOM) (2020). Linee Guida AIOM: Melanoma Cutaneo.

[7] Sklenarova H., Krümpelmann A., Haun M.W., Friederich H., Huber J., Thomas M., Winkler E.C., Herzog W., Hartmann M. (2015). When Do We Need to Care about the Caregiver? Supportive Care Needs, Anxiety, and Depression among Informal Caregivers of Patients with Cancer and Cancer Survivors. Cancer.

[8] Rostami M., Abbasi M., Soleimani M., Moghaddam Z.K., Zeraatchi A. (2023). Quality of Life among Family Caregivers of Cancer Patients: An Investigation of SF-36 Domains. BMC Psychol..

[9] Johansen S., Cvancarova M., Ruland C. (2018). The Effect of Cancer Patients’ and Their Family Caregivers’ Physical and Emotional Symptoms on Caregiver Burden. Cancer Nurs..

[10] Moher D., Liberati A., Tetzlaff J., Altman D.G., The PRISMA Group (2009). Preferred Reporting Items for Systematic Reviews and Meta-Analyses: The PRISMA Statement. PLoS Med..

[11] Tricco A.C., Lillie E., Zarin W., O’Brien K.K., Colquhoun H., Levac D., Moher D., Peters M.D.J., Horsley T., Weeks L. (2018). PRISMA Extension for Scoping Reviews (PRISMA-ScR): Checklist and Explanation. Ann. Intern. Med..

[12] PROSPERO International Prospective Register of Systematic Reviews. https://www.crd.york.ac.uk/prospero/.

[13] Mancini J., Baumstarck-Barrau K., Simeoni M.-C., Grob J.-J., Michel G., Tarpin C., Loundou A.-D., Lambert A., Clément A., Auquier P. (2011). Quality of Life in a Heterogeneous Sample of Caregivers of Cancer Patients: An In-Depth Interview Study: Caregiver-Patient Relationship and Quality of Life. Eur. J. Cancer Care.

[14] Weaver R., O’Connor M., Halkett G.K.B., Carey Smith R. (2021). The Unmet Needs of Carers of Patients Diagnosed with Sarcoma: A Qualitative Study. Psycho-Oncology.

[15] Fox J.A., Rosenberg J., Ekberg S., Langbecker D. (2020). Palliative Care in the Context of Immune and Targeted Therapies: A Qualitative Study of Bereaved Carers’ Experiences in Metastatic Melanoma. Palliat. Med..

[16] Thompson J.R., Fu H., Saw R.P.M., Sherman K.A., Beedle V., Atkinson V., Boyle F., O’Sullivan N.A., Martin L.K., Bartula I. (2023). Supportive Care Needs in Australian Melanoma Patients and Caregivers: Results from a Quantitative Cross-Sectional Survey. Qual. Life Res..

[17] Marshall-McKenna R., Kotronoulas G., Kokoroskos E., Granados A.G., Papachristou P., Papachristou N., Collantes G., Petridis G., Billis A., Bamidis P.D. (2023). A Multinational Investigation of Healthcare Needs, Preferences, and Expectations in Supportive Cancer Care: Co-Creating the LifeChamps Digital Platform. J. Cancer Surviv..

[18] Makady A., Kalf R.R.J., Ryll B., Spurrier G., De Boer A., Hillege H., Klungel O.H., Goettsch W. (2018). Social Media as a Tool for Assessing Patient Perspectives on Quality of Life in Metastatic Melanoma: A Feasibility Study. Health Qual. Life Outcomes.

[19] Milne D., Hyatt A., Billett A., Gough K., Krishnasamy M. (2020). Exploring the Experiences of People Treated With Immunotherapies for Advanced Melanoma and Those Caring for Them: “Real-World” Data. Cancer Nurs..

[20] Shilling V., Starkings R., Jenkins V., Fallowfield L. (2017). The Pervasive Nature of Uncertainty—A Qualitative Study of Patients with Advanced Cancer and Their Informal Caregivers. J. Cancer Surviv..

[21] Aguiar-Ibáñez R., McQuarrie K., Jayade S., Penton H., DiGiovanni L., Raina R., Heisen M., Martinez A. (2025). Impact of Recurrence on Employment, Finances, and Productivity for Early-Stage Cancer Patients and Caregivers: US Survey. Future Oncol..

[22] Boulanger M.C., Falade A.S., Hsu K., Sommer R.K., Zhou A., Sarathy R., Lawrence D., Sullivan R.J., Traeger L., Greer J.A. (2025). Patient and Caregiver Experience with the Hope and Prognostic Uncertainty of Immunotherapy: A Qualitative Study. JCO Oncol. Pract..

[23] Papanikolaou E.S., Sampogna F., Fania L., Di Lella G., Panebianco A., Abeni D., Ricci F. (2022). Quality of Life in Caregivers of Melanoma Patients. Eur. J. Dermatol..

[24] Muliira J.K., Kizza I.B., Nakitende G. (2019). Roles of Family Caregivers and Perceived Burden When Caring for Hospitalized Adult Cancer Patients: Perspective from a Low-Income Country. Cancer Nurs..

[25] Tan J.D., Butow P.N., Boyle F.M., Saw R.P.M., O’Reilly A.J. (2014). A Qualitative Assessment of Psychosocial Impact, Coping and Adjustment in High-Risk Melanoma Patients and Caregivers. Melanoma Res..

[26] Yabroff K.R., Kim Y. (2009). Time Costs Associated with Informal Caregiving for Cancer Survivors. Cancer.

[27] Kim Y., Wellisch D.K., Spillers R.L., Crammer C. (2007). Psychological Distress of Female Cancer Caregivers: Effects of Type of Cancer and Caregivers’ Spirituality. Support. Care Cancer.

[28] Pearlin L.I., Mullan J.T., Semple S.J., Skaff M.M. (1990). Caregiving and the Stress Process: An Overview of Concepts and Their Measures. Gerontologist.

[29] Lazarus R.S., Folkman S. (1984). Stress, Appraisal, and Coping.

[30] CNEL (2024). The Social Value of the Caregiver. A Pathway for Quantifying and Identifying the Emerging Profile of Individuals Who Care for Family Members. An Initial Survey.

[31] Ng J.H.Y., Luk B.H.K., Lee N.P.M. (2023). Gender Differences in Cancer Spousal Caregiving: A Systematic Review. Palliat. Support. Care.

[32] Gaugler J.E., Given W.C., Linder J., Kataria R., Tucker G., Regine W.F. (2008). Work, Gender, and Stress in Family Cancer Caregiving. Support. Care Cancer.

[33] Longo C.J., Fitch M.I., Banfield L., Hanly P., Yabroff K.R., Sharp L. (2020). Financial Toxicity Associated with a Cancer Diagnosis in Publicly Funded Healthcare Countries: A Systematic Review. Support. Care Cancer.

[34] Sadigh G., Switchenko J., Weaver K.E., Elchoufi D., Meisel J., Bilen M.A., Lawson D., Cella D., El-Rayes B., Carlos R. (2022). Correlates of Financial Toxicity in Adult Cancer Patients and Their Informal Caregivers. Support. Care Cancer.

[35] Wittenberg E., Buller H., Ferrell B., Koczywas M., Borneman T. (2017). Understanding Family Caregiver Communication to Provide Family-Centered Cancer Care. Semin. Oncol. Nurs..

[36] Washington K.T., Craig K.W., Parker Oliver D., Ruggeri J.S., Brunk S.R., Goldstein A.K., Demiris G. (2019). Family Caregivers’ Perspectives on Communication with Cancer Care Providers. J. Psychosoc. Oncol..

[37] Lazor T., Rinaldo E., Cyr A., Degeer I. (2019). The Melanoma Patient Exchange: Insights from a Supportive Group Intervention for Melanoma Patients and their Caregivers. Soc. Work. Groups.

[38] Falade A.S., Boulanger M.C., Hsu K., Sarathy R., Fadden R., Reynolds K.L., Traeger L., Temel J.S., Greer J.A., Petrillo L.A. (2024). Learning About and Living with Toxicity: A Qualitative Study of Patients Receiving Immune Checkpoint Inhibitors for Melanoma or Lung Cancer and Their Caregivers. Support. Care Cancer.

[39] Aaronson N.K., Ahmedzai S., Bergman B., Bullinger M., Cull A., Duez N.J., Filiberti A., Flechtner H., Fleishman S.B., de Haes J.C.J.M. (1993). The European Organization for Research and Treatment of Cancer QLQ-C30: A quality-of-life instrument for use in international clinical trials in oncology. J. Natl. Cancer Inst..

[40] Girgis A., Lambert S., Lecathelinais C. (2011). The supportive care needs survey for partners and caregivers of cancer survivors: Development and psychometric evaluation. Psycho-Oncology.

[41] Henry J.D., Crawford J.R. (2005). The short-form version of the Depression Anxiety Stress Scales (DASS-21): Construct validity and normative data in a large non-clinical sample. Br. J. Clin. Psychol..

[42] Grov E.K., Fosså S.D., Tønnessen A., Dahl A.A. (2006). The caregiver reaction assessment: Psychometrics, and temporal stability in primary caregivers of Norwegian cancer patients in late palliative phase. Psycho-Oncology.

[43] McNair D.M., Lorr M., Droppleman L.F. (1992). Revised Manual for the Profile of Mood States.

[44] Basra M.K.A., Sue-Ho R., Finlay A.Y. (2007). The Family Dermatology Life Quality Index: Measuring the secondary impact of skin disease. Br. J. Dermatol..

[45] Sherbourne C.D., Stewart A.L. (1991). The MOS social support survey. Soc. Sci. Med..

[46] Schwarzer R., Jerusalem M., Weinman J., Wright S., Johnston M. (1995). Generalized Self-Efficacy Scale. Measures in Health Psychology: A User’s Portfolio. Causal and Control Beliefs.

[47] Carney S., Koetters T., Cho M., West C., Paul S.M., Dunn L., Aouizerat B.E., Dodd M., Cooper B., Lee K. (2011). Differences in sleep disturbance parameters between oncology outpatients and their family caregivers. J. Clin. Oncol..

[48] Lee K.A., Hicks G., Nino-Murcia G. (1991). Validity and reliability of a scale to assess fatigue. Psychiatry Res..

[49] Radloff L.S. (1977). The CES-D Scale: A Self-Report Depression Scale for Research in the General Population. Appl. Psychol. Meas..

[50] Elmståhl S., Malmberg B., Annerstedt L. (1996). Caregiver’s burden of patients 3 years after stroke assessed by a novel caregiver burden scale. Arch. Phys. Med. Rehabil..

[51] Peterman A.H., Fitchett G., Brady M.J., Hernandez L., Cella D. (2002). Measuring spiritual well-being in people with cancer: The Functional Assessment of Chronic Illness Therapy–Spiritual Well-Being Scale (FACIT-Sp). Ann. Behav. Med..

[52] Reilly M.C., Zbrozek A.S., Dukes E.M. (1993). The validity and reproducibility of a work productivity and activity impairment instrument. Pharmacoeconomics.

