# Supplementary material for: Impact on Quality of Life and Psychological Dimensions in Caregivers of Melanoma and Sarcoma Patients: A Scoping Review
Source: Cancers (Basel). 2026 Mar 2;18(5):809. doi: 10.3390/cancers18050809 (PMC12984831; doi:10.3390/cancers18050809)
Supplement: Supplementary file 1 [file cancers-18-00809-s001.zip › Table S7. Instruments and Methods Used to Evaluate Caregiver Quality of Life and Psychological Distress across the articles.pdf]

Assessment tools	Construct assessed	Domains/Subscales	QoL or Psychological Domains	Number of items	Response format	Mode of administration	Score range	Interpretation	Target population	Validity (Cronbach's alpha)	Psychometric informations in caregiver populations	Studies using the instrument included in the revision paper	Population on which the instrument was administered	Clinical context in wich instrument was used (disease)	Clinical context in wich instrument was used (stage of disease)	Validation article reference used in the articles included in the revision paper
European Organisation for Research and Treatment of Cancer Quality of Life Questionnaire (EORTC QLQ-C30)	Helath Related Quality of Life	5 functional scales (physical, role, cognitive, emotional, social), 3 general symptom scales (fatigue, nausea/vomiting, pain), 6 single symptom scales (dyspnea, insomnia, appetite loss, constipation, diarrhoea, financial impact)	QoL	30	4-point likert scale (1-4) for functional and symptom scales; 6-point scale (1-7) for global health	Self-administered questionnaire	0–100 per scale	Higher scores indicate better functioning for functional scales and worse symptoms for symptom scales; global QoL higher indicate better QoL	Adult cancer patients	>0.70	Not validated on caregivers population	Thompson J. R. [21]	Caregivers	Melanoma	Early and advanced stage	Aaronson, N. K.; Ahmedzai, S.; Bergman, B.; Bullinger, M.; Cull, A.; Duez, N. J.; Filiberti, A.; Flechtner, H.; Fleishman, S. B.; de Haes, J. C. J. M.; Kaasa, S.; Klee, M.; Osoba, D.; Razavi, D.; Rofe, P. B.; Schraub, S.; Sneeuw, K.; Sullivan, M.; Takeda, F. The European Organization for Research and Treatment of Cancer QLQ-C30: A quality-of-life instrument for use in international clinical trials in oncology. *J. Natl. Cancer Inst.* **1993**, 85(5), 365–376. doi:10.1093/jnci/85.5.365.
Supportive Care Needs Survey for Partners and Caregivers (SCNS-P&C)	Unmet supportive care needs of partners and caregivers	Health-care service needs<br>Psychological and emotional needs<br>Work and social needs<br>Information needs	Both	44	5-point Likert scale (1-5)	Self-administered questionnaire	1–5 per item	Higher scores indicate greater unmet needs	Partners and caregivers of cancer survivors	0.88 – 0.94	Validated on caregivers population	Thompson J. R. [21]	Caregivers	Melanoma	Various stages	Girgis, A.; Lambert, S.; Lecathelinais, C. The supportive care needs survey for partners and caregivers of cancer survivors: development and psychometric evaluation. *Psycho-Oncology* **2011**, 20(4), 387–393. doi: 10.1002/pon.1740.
Depression, Anxiety and Stress Scale - 21 Items (DASS-21)	Depression, anxiety, and stress	Depression, anxiety, stress	Psychological Domains	21	4-point Likert scale (0-3)	Self-administered questionnaire	Scores summed per subscale	Higher scores indicate greater symptom severity	General/Clinical population	0.88 depression, 0-82 anxiety, 0.92 stress, 0.93 total scale	Not validated on caregivers population	Thompson J. R. [21]	Caregivers	Melanoma	Various stages	Henry, J. D.; Crawford, J. R. The short-form version of the Depression Anxiety Stress Scales (DASS-21): construct validity and normative data in a large non-clinical sample. *Br. J. Clin. Psychol.* **2005**, 44(Pt 2), 227–239. doi:10.1348/014466505X29657.
Caregiver Reaction Assessment (CRA)	Caregiving burden	Self Esteem, Lack of Family Support, Impact on Finances, Impact on Daily Schedule, Impact on Health	Psychological Domains	24	5-point Likert scale (1-5)	Self-administered questionnaire	Scores summed per subscale	Higher scores indicate greater burden except for Self Esteem (higher = positive)	General/Clinical population	0.57-0-85, 0.74 total	Validated on caregivers population	Johansen S. [9]	Caregivers	Melanoma	Stage IV	Grov, E. K.; Fosså, S. D.; Tønnessen, A.; Dahl, A. A. The caregiver reaction assessment: psychometrics, and temporal stability in primary caregivers of Norwegian cancer patients in late palliative phase. *Psycho‑Oncology* **2006**, 15(6), 517–527. doi:10.1002/pon.987.
Profile of Mood States - Short Form (POMS-SF)	Mood	Tension or Anxiety, Depression or Dejection, Anger or Hostility, Vigor or Activity, Fatigue or Inertia, Confusion or Bewilderment	Psychological Domains	37	5-point Likert scale (0-4)	Self-administered questionnaire	Scores summed per subscale	Higher scores indicate greater intensity of mood state	General population	0.85-0.95	Validated on caregivers population	Kim Y. [25]	Caregivers	Breast, kidney, lung, non-Hodgkin' slymphoma, melanoma, ovarian cancer.	-	McNair, D. M.; Lorr, M.; Droppleman, L. F. Profile of Mood States, Revised ed.; EdITS/Educational and Industrial Testing Service: San Diego, CA, 1992.
Family Dermatology Life Quality Index (FDLQI)	Impact of skin diseases on family quality of life	-	QoL	10	4-point Likert scale (0-3)	Self-administered questionnaire	0–30 total	Higher scores indicate greater negative impact on family members' quality of life	Family members of dermatology patients	0.88	Validated on caregivers population	Papanikolaou E.S. [19]	Caregivers	Melanoma	All stages	Basra, M. K. A.; Sue‑Ho, R.; Finlay, A. Y. The Family Dermatology Life Quality Index: measuring the secondary impact of skin disease. *Br. J. Dermatol.* **2007**, 156(3), 528–538. doi:10.1111/j.1365‑2133.2006.07617.x.
Medical Outcomes Study Social Support Survey (MOS-SSS)	Perceived social support	Emotional/Informational support<br>Tangible support<br>Affectionate support<br>Positive social interaction support	Psychological Domains	19	5-point Likert scale (1-5)	Self-administered questionnaire	0–100 per subscale	Higher scores indicate greater perceived support	Patients with chronic illnesses	0.91	Not validated on caregivers population	Johansen S. [9]	Caregivers	Breast, prostate, melanoma, myelomatose, lymphoma, headneckcancers	-	Sherbourne, C. D.; Stewart, A. L. The MOS social support survey. *Soc. Sci. Med.* **1991**, 32(6), 705–714. doi:10.1016/0277‑9536(91)90150‑B.
General Self Efficacy Scale (GSES)	General self-efficacy	-	Psychological Domains	10	4-point likert scale (1-4)	Self-administered questionnaire	10-40 total	Higher scores indicate more self-efficacy	General population	0.76 – 0.90	Not validated on caregivers population	Johansen S. [9]	Caregivers	Breast, prostate, melanoma, myelomatose, lymphoma, headneckcancers	-	Schwarzer, R.; Jerusalem, M.; Weinman, J.; Wright, S.; Johnston, M. Generalized Self‑Efficacy Scale. In Measures in Health Psychology: A User's Portfolio. Causal and Control Beliefs; NFER-NELSON: Windsor, UK, 1995.
General Sleep Disturbance Scale (GSDS)	Incidence and nature of sleep disturbance	Quality of sleep, Quantity of sleep, Sleep onset latency, Midsleep awakenings, Early awakenings, Medications for sleep, Excessive daytime sleepiness	QoL	21	8-point Likert-type scale (0-7)	Self-administered questionnaire	0-147 total	Higher total and subscale scores indicated higher levels of sleep disturbance. Subscales scores of $\geq$ 3 and a GSDS total score of $\geq$ 43 indicates a significant level of sleep disturbance	General population	0.79	Validated on caregivers population	Johansen S. [9]	Caregivers	Breast, prostate, melanoma, myelomatose, lymphoma, headneckcancers	-	Carney, S.; Koetters, T.; Cho, M.; West, C.; Paul, S. M.; Dunn, L.; Aouizerat, B. E.; Dodd, M.; Cooper, B.; Lee, K.; Wara, W.; Swift, P.; Miaskowski, C. Differences in sleep disturbance parameters between oncology outpatients and their family caregivers. *J. Clin. Oncol.* **2011**, 29 (8), 1001–1006. doi:10.1200/JCO.2010.30.9104.
Lee Fatigue Scale (LFS)	Fatigue severity	Fatigue subscale, Energy subscale	QoL	18	Visual Analog Scale	Self-administered questionnaire	0-30, 0-50, 0-130	Higher scores indicate higher levels of perceived fatigue and energy	General/Clinical population	0.94 – 0.96	Validated on caregivers population	Johansen S. [9]	Caregivers	Breast, prostate, melanoma, myelomatose, lymphoma, headneckcancers	-	Lee, K. A.; Hicks, G.; Nino‑Murcia, G. Validity and reliability of a scale to assess fatigue. *Psychiatry Res.* **1991**, 36(3), 291–298. doi:10.1016 /0165‑1781(91)90027‑M.
Center for Epidemiologic Studies Depression Scale (CES-D)	Presence and severity of depressive symptoms	-	Psychological Domains	20	4-point Likert scale (0-3)	Self-administered questionnaire	0-60	Higher scores indicate greater depressive symptoms (cut-off = 16)	General population	<0.85	Validated on caregivers population	Johansen S. [9]	Caregivers	Breast, prostate, melanoma, myelomatose, lymphoma, headneckcancers	-	Radloff, L.S. The CES-D Scale: A Self-Report Depression Scale for Research in the General Population. *Appl. Psychol. Meas.* **1977**, 1, 385–401. https://doi.org/10.1177/014662167700100306
Caregiver Burden Scale (CBS)	Caregiver burden	General strain, Isolation, Disappointment, Emotional Involment, Environment	Both	22	5-point Likert scale (0-4)	Self-administered questionnaire	0-88	Higher scores indicate greater caregiver burden (0-20 = little/no burden, 21-40 = mild/moderate burden, 41-60 = moderate/severe burden, 61-88 = severe burden)	Caregiver population	0.70 – 0.87 (except for environment)	Validated on caregivers population	Muliira J.K. [17]	Caregivers	Kaposi's sarcoma, prostate carcinoma, leukemia, pancreatic cancer, esophageal cancer, bone cancer, seminoma, hepatocarcinoma, colorectal cancer,breast cancer	-	Elmståhl, S.; Malmberg, B.; Annerstedt, L. Caregiver's burden of patients 3 years after stroke assessed by a novel caregiver burden scale. *Arch. Phys. Med. Rehabil.* **1996**, 77, 177–182. doi:10.1016/S0003-9993(96) 90164-1.
Pearlin Role Overload Measure (Pearlin ROM)	Stress	-	Psychological Domains	4	4-point likert scale (1-4)	Self-administered questionnaire	4-16	Higher scores indicate greater overload	Caregiver population	0.80	Validated on caregivers population	Kim Y. [25]	Caregivers	Breast, kidney, lung, non-Hodgkin' slymphoma, melanoma, ovarian cancer.	-	Pearlin, L. I.; Mullan, J. T.; Semple, S. J.; Skaff, M. M. Caregiving and the stress process: An overview of concepts and their measures. *Gerontologist* **1990**, 30, 583–594. doi:10.1093/geront/30.5.583.
FACIT-Sp12	Spritual Well-being	Spritual Well-being, Meaning, Peace, Faith	QoL	12	5-point Likert scale (0-4)	Self-administered questionnaire	0-48	Higher scores better QoL Spritual Well-being	Clinical Population	0.89	Validated on caregivers population	Kim Y. [25]	Caregivers	Breast, kidney, lung, non-Hodgkin' slymphoma, melanoma, ovarian cancer.	-	Peterman, A. H.; Fitchett, G.; Brady, M. J.; Hernandez, L.; Cella, D. Measuring spiritual well-being in people with cancer: the Functional Assessment of Chronic Illness Therapy–Spiritual Well-Being Scale (FACIT-Sp). *Ann. Behav. Med.* **2002**, 24, 49–58. doi:10.1207 /S15324796ABM2401_06.
Work Productivity and Activity Impairment: Caregiver (WPAI:CG)	Work and activity impairment	Absenteism, Presenteism, Overall Work Impairment, Activity Impairment	QoL	6	1 item yes/no, 3 open item, 2 visual analog scale (0-10)	Self-administered questionnaire	0-100%	Higher scores indicate less interference	Patients with health condition	<0.70	Validated on caregivers population	Aguiar-Ibanez R. [15]	Caregivers	Bladder, gastric, head and neck, non–small cell lung,renal cell, triple-negative breastcancers, melanoma	-	Reilly, M. C.; Zbrozek, A. S.; Dukes, E. M. The validity and reproducibility of a work productivity and activity impairment instrument. *Pharmacoeconomics* **1993**, 4(5), 353–365. doi:10.2165/00019053-199304050-00006.
