# Supplementary material for: Impact on Quality of Life and Psychological Dimensions in Caregivers of Melanoma and Sarcoma Patients: A Scoping Review
Source: Cancers (Basel). 2026 Mar 2;18(5):809. doi: 10.3390/cancers18050809 (PMC12984831; doi:10.3390/cancers18050809)
Supplement: Supplementary file 1 [file cancers-18-00809-s001.zip › Table S5. Baseline characteristics of the included studies - study details and sample characteristics.pdf]

Author	Title	Type of pub	Year	Study design	Country	Number of patients (caregivers)	Age of patients (mean, SD or range)	Gender of patients	Relationship	Type of cancer	Stage of cancer
Mancini J. [13]	Quality of life in a heterogeneous sample of caregivers of cancer patients: An in‑depth interview study.	Article	2011	Qualitative Research	France	77 (17 melanoma)	48,1 (18-81) (melanoma 55.8 (32-70))	F:49 M:28 Melanoma F:8 M:9	2 parent, 12 spouse, 2 child, 1 friend	breast cancer, melanoma, paediatric haematology	II, III (for melanoma)
Weaver R. [16]	The unmet needs of carers of patients diagnosed with sarcoma: A qualitative study.	Article	2021	Qualitative Research	Australia	33	51 (range 22-66)	F:26 M:12	15 mother, 4 father, 11 spouse, 2 brother, 1 daughter	sarcoma	-
Fox J.A. [27]	Palliative care in the context of immune and targeted therapies: A qualitative study of bereaved carers' experiences in metastatic melanoma.	Article	2020	Qualitative Research	Australia	20	30 to 39 years 1 (5%)<br>40 to 49 years 4 (20%)<br>50 to 59 years 3 (15%)<br>60 to 69 years 9 (45%)<br>70 to 79 years 3 (15%)	F:16 M:4	16 partner, 2 sibling, 2 child	metastatic melanoma	-
Thompson J.R. [21]	Supportive care needs in Australian melanoma patients and caregivers: results from a quantitative cross-sectional survey.	Article	2023	Quantitative Cross-sectional Survey	Australia	37	55 (12)	F:32 M:5	29 partner, 8 immediate family member	melanoma	early and advanced stage
Marshall-McKenna R. [24]	A multinational investigation of healthcare needs, preferences, and expectations in supportive cancer care: Co-creating the LifeChamps digital platform.	Article	2022	Descriptive, Cross-sectional, Multi-method study.	Greece, Spain, Sweden, United Kingdom	23	50,3 (14,8)	F:9 M:4	10 daughter, 8 spouse/partner, 3 other, 1 sister in law, 1 son	breast cancer, prostate cancer, melanoma	cancer survivor
Makady A. [23]	Social media as a tool for assessing patient perspectives on quality of life in metastatic melanoma: A feasibility study.	Article	2018	Survey	Belgium, France, Netherlands, Romania, United Kingdom, other	17	<40 (6%)<br>40 to 49 years (12%)<br>50 to 59 years (24%)<br>60 to 69 years (29%)<br>70 to 79 years (24%)<br>80+ (6%)	F:17 M:9	-	melanoma	all stage
Johansen S. [9]	The effect of cancer patients' and their family caregivers' physical and emotional symptoms on caregiver burden.	Article	2018	Cross-sectional quantitative research	Norway	281 (42 head neck and skin)	≤50 (96)<br>> 50 (185)	F:149 M:132	227 spouse/partner, 45 family member, 9 other family member	breast, prostate, melanoma, myelomatose, lymphoma, head-neck cancers	-
Milne D. [14]	Exploring the experiences of people treated with immunotherapies for advanced melanoma and those caring for them: 'Real-world' data.	Article	2020	Qualitative Research, Cross-sectional	Australia	9	49 (range 32-65)	F:8 M:1	-	melanoma	IV
Shilling V. [18]	The pervasive nature of uncertainty—A qualitative study of patients with advanced cancer and their informal caregivers.	Article	2017	Qualitative Research	United Kingdom	8	53 (36-70)	F:5 M:3	spouse/partner	ovarian, melanoma, lung cancer	III, IV
Aguiar-Ibanez R. [15]	Impact of recurrence on employment, finances, and productivity for early-stage cancer patients and caregivers: US survey	Article	2024	Cross-sectional, Non-interventional, Online survey	United States of America	100 (17 melanoma)	51.7	F:71 M:28 Non binary:1	48 spouse/significant other, 24 son/daughter, 10 parent, 9 friend/neighbour, 9 sibling	bladder, gastric, head and neck, non–small cell lung, renal cell, triple-negative breast cancers, melanoma	-
Boulanger M.C. [22]	Patient and caregiver experience with the hope and prognostic uncertainty of immunotherapy: A qualitative study	Article	2024	Qualitative Research	United States of America	10 (7 melanoma)	77 (31-80)	F:5 M:4 Missing: 1	8 Spouse, 1 sibling, 1 other family member	melanoma, NSCLC	III, IV
Papanikolaou E.S. [19]	Quality of life in caregivers of melanoma patients	Article	2022	-	Italy	120	<40 (36,7%)<br>40-54 (40,8%)<br>> 55 (22,5%)	F:51 M:69	50 son/daughter, 23 partner, 27 brother/sister, 20 other	melanoma	all stage
Muliira J.K. [17]	Roles of family caregivers and perceived burden when caring for hospitalized adult cancer patients: Perspective from a low-income country	Article	2018	Cross sectional, Descriptive design	Africa (Uganda)	168	36 (12.7)	F:128 M:40	46 spouse, 122 not spouse	kaposi's sarcoma, prostate carcinoma, leukemia, pancreatic cancer, esophageal cancer, bone cancer, seminoma, hepatocarcinoma, colorectal cancer, breast cancer	-
Tan J.D. [26]	A qualitative assessment of psychosocial impact, coping and adjustment in high-risk melanoma patients and caregivers	Article	2014	Qualitative Research	Australia	14	57 (11)	F:11 M:3	8 partner, 1 parent, 1 child, 2 friend, 2 other	melanoma	III
Yabroff K.R. [20]	Time costs associated with informal caregiving for cancer survivors	Article	2009	Qualitative research	United States of America	688 (73 bladder, skin and uterine)	<24 (1.5%)<br>25-34 (5.8%)<br>35-44 (12.4%)<br>45-54 (32.4%)<br>55-64 (26.2%)<br><65 (19%)<br>Missing data n=19 (2.8%)	F:450 M:238	451 spouse/partner, 111 child/child-in-law, 29 parent, 57 sibling, 26 friend, 14 other	bladder, breast, colorectal, kidney, lung, melanoma of the skin, ovarian, prostate, or uterine cancer, non-Hodgkins lymphoma (NHL)	-
Kim Y. [25]	Psychological distress of female cancer caregivers: Effects of type of cancer and caregivers' spirituality	Article	2007	Qualitative research	United States of America	1635 (7 melanoma)	48.35 (14.52)	F:1068 M:567	28 mother, 60 sister, 110 daughter, 21 friend, 8 daughter-in-law,<br>6 other in-law, 7 partner, 12 other (just female)	breast, kidney, lung, non-Hodgkin's lymphoma, melanoma, ovarian cancer.	-
