# Supplementary material for: Impact on Quality of Life and Psychological Dimensions in Caregivers of Melanoma and Sarcoma Patients: A Scoping Review
Source: Cancers (Basel). 2026 Mar 2;18(5):809. doi: 10.3390/cancers18050809 (PMC12984831; doi:10.3390/cancers18050809)
Supplement: Supplementary file 1 [file cancers-18-00809-s001.zip › Table S4. Data extraction form.pdf]

DATA EXTRACTION FORM

**STUDY DETAILS**	
Authors	
Title	
Type of publication	• Article<br>• Book chapter
Year of publication	
Type of study	• Randomized Controlled Trials<br>• Quasi-Experimental Studies<br>• Cohort Studies<br>• Case-Control Studies<br>• Cross-Sectional Studies<br>• Case Reports/Series<br>• Qualitative Studies
**SAMPLE CHARACTERISTICS**	
**N**	
Age	
Gender	
Relationship	
Country	
Type of cancer	
Phase of cancer	
Educational level (when available)	
**MEASURES**	
Type of assessment	• Qualitative<br>• Quantitative
Qol tools (for caregiver)	
Qol dimensions measured	
Psychological tools (for caregiver)	
Psychological dimension measured	
**MAJOR FINDINGS**	
