# Supplementary material for: Impact on Quality of Life and Psychological Dimensions in Caregivers of Melanoma and Sarcoma Patients: A Scoping Review
Source: Cancers (Basel). 2026 Mar 2;18(5):809. doi: 10.3390/cancers18050809 (PMC12984831; doi:10.3390/cancers18050809)
Supplement: Supplementary file 1 [file cancers-18-00809-s001.zip › Table S6. Main findings of the included studies on quality of life and psychological dimensions in caregivers of patients with melanoma and or sarcoma.pdf]

Author	Assessment Type	Health-Related Quality of Life (HRQoL) Measures	HRQoL Dimensions	Psychological Measures	Psychological Dimensions	Major Findings
Mancini J. [13]	Qualitative	Semi-structured Interviews	Leisure and daily activities; occupation and financial issues; physical well-being; relationship with healthcare professionals; relationship with family and friends; patient–caregiver relationship; relationships with institutional caregivers	Semi-structured Interviews	Psychological well-being: anxiety, fear, stress, sadness, depression, insecurity, and perceived injustice or unfairness; relationship with the patient: intimacy, communication, and emotional dynamics	In line with previous literature, the study found that caregiving was primarily associated with a significant psychological burden, even in cases involving patients with favorable prognoses, while serious physical consequences were rare. The findings highlight the need for a standardized assessment of caregivers' quality of life, supplemented by specific modules for cancer and relationships.
Weaver R. [16]	Qualitative	Semi-structured Interviews	Support with Medical Aspects of Caregiving: Medical tasks and care-related support; Need for Information about the Patient: Access to patient-related information; Financial Impact: Economic burden of caregiving	Semi-structured Interviews	Psychological Support for Caregivers and Family: Emotional and psychological support needs of caregivers and family members	Caregivers of sarcoma patients report that the patients' needs, which also affect them, are not being met in several areas: medical, informational, psychosocial and financial. Many caregivers have experienced psychological distress but have struggled to seek support, prioritising the needs of their patients, which has had a negative impact on their quality of life and ability to provide care. It is therefore necessary to develop support programmes tailored to caregivers, including financial assistance and support groups.
Fox J.A. [27]	Qualitative	-	-	Semi-structured Interviews (Grounded Theory)	Psychological dimensions: hope-related expectations, treatment continuation despite uncertain benefit, unmet informational needs, delayed integration of palliative care, and lack of end-of-life planning	The investigation reveals that care providers encounter a variety of difficulties, especially when it comes to acquiring clear infomrations from medical practitioners, for example during the transition to end-of-life care. Several factors contribute to caregivers' feelings of overwhelm and stress, including understanding complex prognostic data, navigating the debate between palliative and continuing care, and a lack of preparation for the end of life of the person they are caring for. These findings highlight the ongoing need for information, psychosocial support and preparation, and emphasise the importance of educational initiatives and improved communication between doctors, patients and caregivers to alleviate the burden on caregivers.
Thompson J.R. [21]	Mixed-Methods	European Organisation for Research and Treatment of Cancer Quality of Life Questionnaire (EORTC QLQ-C30); Supportive Care Needs Survey Partners&Caregiver (SCNS-P&C)	Caregiver quality of life: healthcare-related needs, work and social functioning, and global health	Depression, Anxiety and Stress Scale (DASS-21)	Psychological distress: anxiety, depression, stress, and distress related to unmet information needs	The study revealed that Australian melanoma patients and their caregivers have significant unmet psychological and emotional needs. The findings highlight the need for a standardized assessment of caregivers' quality of life, supplemented by specific modules on cancer and relationships. Focusing on these aspects would help facilitate the resolution of the issues identified.
Marshall-McKenna R. [24]	Mixed-Methods	Online Survey	Pervasive Uncertainty: Widespread and ongoing uncertainty across care trajectories	Online Survey	Psychological Needs: Need for psychological support; Relationship with Institutions and Information Needs: Interaction with institutions and access to information.	The analysis conducted by the authors shows that cancer survivors and their family caregivers report dissatisfaction with the lack of clear information and ongoing support throughout the cancer journey. Given these gaps, follow-up services and targeted psychological support are essential to address these shortcomings.
Makady A. [23]	Quantitative	Qualitative Interviews	Family and Social Relationships; Emotional Burden: Fear and concerns.	/	/	This study explored the feasibility of using social media to assess the perspectives of patients and their caregivers on health-related quality of life (HRQoL). The research found that caregivers prioritized the ability to cope with manageable adverse events, to be capable, and to be free from pain, while patients prioritized family, emphasizing the importance of leading a normal life and enjoying life. Some also emphasized the importance of not neglecting career-related aspects.
Johansen S. [9]	Quantitative	Caregiver Reaction Assessment (CRA); Medical Outcomes Study Social Support Survey (MOS-SSS); General Self-Efficacy Scale (GSES); General Sleep Disturbance Scale (GSDS)	Fatigue; Sleep Disturbance; Symptom Distress	Caregiver Reaction Assessment (CRA);<br>Lee Fatigue Scale (LFS); Center for Epidemiologic Studies Depression Scale (CES-D)	Psychological and social outcomes: fatigue, depression, sense of self-efficacy, stress and emotional distress, perceived social support	The research found that a significant burden on caregivers is associated with depression, fatigue, sleep disturbances, low self-esteem and limited social support in patients. The survey found that female patients and caregivers experienced a greater burden than men. These findings emphasise the importance of including caregivers in the cancer care pathway.
Milne D. [14]	Qualitative	Qualitative Interviews	Economic and physical burden: financial toxicity and fatigue	Qualitative Interviews	Treatment-related uncertainty and associated anxiety	This research focuses on patients receiving immunotherapy for stage IV melanoma and their caregivers, who report experiencing a reduced quality of life due to treatment-related toxicities, stress, financial hardship and fatigue. Key challenges include uncertainty and an increased burden of caregiving responsibilities and side effects. The findings highlight the need for comprehensive preparation, clear information and rapid access to knowledgeable healthcare professionals to support patients and their caregivers.
Shilling V. [18]	Qualitative	Qualitative Interviews	Job and financial implications: concerns related to employment, loss of earnings, and perceived financial position<br>Implications for the future: changes in outlook, realigning priorities, life on hold, opportunities lost, and inability to plan for the future	Qualitative Interviews	Managing uncertainty: control, preservation of or return to normality, hope, and mindset<br>Relationships and communication: patient–caregiver relationship and communication, prevalence of cancer-related conversations, and family dynamics	Patients and caregivers faced significant uncertainty about the future, leading to loss of control over work, finances, family, and retirement. Coping focused on maintaining “normality,” with family well-being prioritized by patients and caregivers feeling their lives “on hold.” Impact varied by age and closeness to the patient, highlighting the need for open communication and targeted support.
Aguiar-Ibanez R. [15]	Qualitative	Online Survey; Work Productivity and Activity Impairment: Caregiver (WPAI: CG)	Work productivity and activity impairment	Online Survey	/	Cancer recurrence has a substantial negative impact on work productivity, employment stability, and financial well-being for both patients and caregivers. These challenges contribute to increased stress, limit the ability to participate in daily activities, and exacerbate the overall burden of caregiving, highlighting the need for targeted support and interventions to mitigate these effects.
Boulanger M.C. [22]	Qualitative	Qualitative Interviews	Disruption of family and occupational roles	Qualitative Interviews	Hope and prognostic uncertainty; overwhelming disappointment among patients without long-term response; conflicting preferences for receiving prognostic information; chronic stress related to ongoing prognostic uncertainty and constant vigilance; perceived lack of control over the course of treatment and the patient’s prognosis	Optimistic expectations were often influenced by oncology teams, but uncertainty and unpredictable long-term treatment responses caused emotional distress and disappointment. Patients and caregivers had differing preferences for prognostic information, underscoring the need for personalized communication strategies.
Papanikolaou E.S. [19]	Quantitative	Family Dermatology Life Quality Index (FDLQI)	Time dedicated to caregiving; impact on work or studies; social activities and leisure time; personal relationships; daily activities; financial aspects; sleep and rest; overall impact on quality of life	Family Dermatology Life Quality Index (FDLQI)	Emotional and psychological well-being; self-perception in the caregiver role	Emotional distress was identified as the primary contributor to caregiver burden on the FDLQI. Burden was higher for son/daughter caregivers and increased with patient age and time since diagnosis, whereas caregiver sex, age, and educational level showed no significant effect.

Muliira J.K. [17]	Quantitative	/	/	Caregiver Burden Scale (CBS)	Psychological dimensions: emotional and psychological well-being; self-perceived role and identity as a caregiver	Caregivers in low-income settings experience high, multidimensional burden, including physical, emotional, and social impacts. Key interventions to alleviate this burden include emotional support, training, and practical assistance such as dedicated nursing care.
Tan J.D. [26]	Qualitative	Semi-structured Phone Interviews	Treatment impact: disruption of daily life, including occupational, social, and family roles; financial impact; caregiver role devaluation	Semi-structured Phone Interviews	Emotional distress; feelings of isolation, helplessness, and lack of support from healthcare systems; coping strategies, including adaptive (support, helpful thinking, meaning-making) and maladaptive (avoidance, suppression)	Patients and caregivers reported significant emotional distress across all disease phases, including shock, anxiety, fear, sadness, and frustration. Caregivers assumed new roles during treatment, leading to feelings of inadequacy, guilt, and being overwhelmed, with some experiencing devaluation of their own struggles. Coping strategies varied by disease phase. Findings highlight the need for routine psychological screening, enhanced communication, and targeted supportive care for both patients and caregivers.
Yabroff K.R. [20]	Quantitative	Survey	Time costs related to caregiving; financial impact	Survey	Emotional support and instrumental support (providing practical help and assistance)	Caregiving for cancer patients imposes substantial time demands, averaging 8.3 hours per day over 13.7 months, with the highest burden for patients with distant disease. About half of caregivers provided emotional, instrumental, tangible, or medical support, highlighting the significant time-related burden and costs in the first two years after diagnosis.
Kim Y. [25]	Qualitative	FACIT-Sp	Spiritual Well-Being	Profile od Mood States (POMS-SF); Pearlin Stress Scale;	Psychological dimensions: caregiving stress; caregiver spirituality; caregiver psychological distress	Caregivers of survivors with nongender-specific cancers experienced higher psychological distress than those caring for gender-specific cancers. Increased caregiving stress and lower spirituality were linked to greater distress, whereas higher spirituality mitigated stress effects, emphasizing the influence of cancer type and personal spiritual resources on caregiver adjustment.
