# Supplementary material for: Impact on Quality of Life and Psychological Dimensions in Caregivers of Melanoma and Sarcoma Patients: A Scoping Review
Source: Cancers (Basel). 2026 Mar 2;18(5):809. doi: 10.3390/cancers18050809 (PMC12984831; doi:10.3390/cancers18050809)
Supplement: Supplementary file 1 [file cancers-18-00809-s001.zip › Table S3. List of excluded article after full reading.pdf]

Authors	Title	Type of pub	Year of pub	Reason for exclusion	Reference/DOI
Lazor T. [37]	The Melanoma Patient Exchange: Insights from a Supportive Group Intervention for Melanoma Patients and their Caregivers.	Article	2019	Excluded due to focus on feasibility and satisfaction of a group intervention rather than caregiver-reported psychological or quality-of-life outcomes.	Lazor, T.; Rinaldo, E.; Cyr, A.; Degeer, I. The Melanoma Patient Exchange: Insights from a Supportive Group Intervention for Melanoma Patients and their Caregivers. *Social Work With Groups* **2019**, 42, 18–28. https://doi.org/10.1080/01609513.2017.1380560
Falade A. [38]	Learning about and living with toxicity: a qualitative study of patients receiving immune checkpoint inhibitors for melanoma or lung cancer and their caregivers.	Article	2024	Excluded because the study primarily focused on patients' experiences with immune checkpoint inhibitor–related toxicity, while caregivers were included only marginally and without dedicated assessment of caregiver psychological outcomes or quality of life.	Falade, A.S.; Boulanger, M.C.; Hsu, K.; Sarathy, R.; Fadden, R.; Reynolds, K.L.; Traeger, L.; Temel, J.S.; Greer, J.A.; Petrillo, L.A. Learning About and Living With Toxicity: A Qualitative Study of Patients Receiving Immune Checkpoint Inhibitors for Melanoma or Lung Cancer and Their Caregivers. *Research Square* **2024**, rs.3.rs-4576328. https://doi.org/10.21203/rs.3.rs-4576328/v1
