# Supplementary material for: Impact on Quality of Life and Psychological Dimensions in Caregivers of Melanoma and Sarcoma Patients: A Scoping Review
Source: Cancers (Basel). 2026 Mar 2;18(5):809. doi: 10.3390/cancers18050809 (PMC12984831; doi:10.3390/cancers18050809)
Supplement: Supplementary file 1 [file cancers-18-00809-s001.zip › Table S2. Full Search Strings.pdf]

Database	Date of last search	Search Strategy	Results (N)
PubMed	21/01/2025	((sarcoma[Title/Abstract]) AND (caregiver[Title/Abstract])) OR ((melanoma[Title/Abstract]) AND (caregiver[Title/Abstract]))<br><br>((sarcoma[Title/Abstract]) AND (spouse[Title/Abstract])) OR ((melanoma[Title/Abstract]) AND (spouse[Title/Abstract]))<br><br>((sarcoma[Title/Abstract]) AND (informal caregiver[Title/Abstract])) OR ((melanoma[Title/Abstract]) AND (informal caregiver[Title/Abstract]))<br><br>((sarcoma[Title/Abstract]) AND (family caregiver[Title/Abstract])) OR ((melanoma[Title/Abstract]) AND (family caregiver[Title/Abstract]))<br>**Tot**	41<br>37<br>2<br>0<br>**80**
Embase	21/01/2025	1# sarcoma:ab,ti AND 'caregiver':ab,ti OR (melanoma:ab,ti AND 'caregiver':ab,ti)<br>2# sarcoma:ab,ti AND 'spouse':ab,ti OR (melanoma:ab,ti AND 'spouse':ab,ti)<br>3# sarcoma:ab,ti AND 'informal caregiver':ab,ti OR (melanoma:ab,ti AND 'informal caregiver':ab,ti)<br>4# sarcoma:ab,ti AND 'family caregiver':ab,ti OR (melanoma:ab,ti AND 'family caregiver':ab,ti)<br>**Tot**	98<br>99<br>6<br>1<br>**204**
PsychINFO	21/01/2025	XB (sarcoma) AND XB (caregiver)<br>XB (melanoma) AND XB (caregiver)<br>XB (sarcoma) AND XB (spouse)<br>XB (melanoma) AND XB (spouse)<br>XB (sarcoma) AND XB (informal caregiver)<br>XB (melanoma) AND XB (informal caregiver)<br>XB (melanoma) AND XB (family caregiver)<br>XB (sarcoma) AND XB (family caregiver)<br>**Tot**	7<br>15<br>2<br>9<br>1<br>3<br>3<br>1<br>**41**
**Total**			325
