# Supplementary material for: Impact on Quality of Life and Psychological Dimensions in Caregivers of Melanoma and Sarcoma Patients: A Scoping Review
Source: Cancers (Basel). 2026 Mar 2;18(5):809. doi: 10.3390/cancers18050809 (PMC12984831; doi:10.3390/cancers18050809)
Supplement: Supplementary file 1 [file cancers-18-00809-s001.zip › Supplementary Files.pdf]

Authors	Title	Type of pub	Year of pub	Reason for exclusion	Reference/DOI
Lazor T. [37]	The Melanoma Patient Exchange: Insights from a Supportive Group Intervention for Melanoma Patients and their Caregivers.	Article	2019	Excluded due to focus on feasibility and satisfaction of a group intervention rather than caregiver-reported psychological or quality-of-life outcomes.	Lazor, T.; Rinaldo, E.; Cyr, A.; Degeer, I. The Melanoma Patient Exchange: Insights from a Supportive Group Intervention for Melanoma Patients and their Caregivers. *Social Work With Groups* **2019**, 42, 18–28. https://doi.org/10.1080/01609513.2017.1380560
Falade A. [38]	Learning about and living with toxicity: a qualitative study of patients receiving immune checkpoint inhibitors for melanoma or lung cancer and their caregivers.	Article	2024	Excluded because the study primarily focused on patients' experiences with immune checkpoint inhibitor–related toxicity, while caregivers were included only marginally and without dedicated assessment of caregiver psychological outcomes or quality of life.	Falade, A.S.; Boulanger, M.C.; Hsu, K.; Sarathy, R.; Fadden, R.; Reynolds, K.L.; Traeger, L.; Temel, J.S.; Greer, J.A.; Petrillo, L.A. Learning About and Living With Toxicity: A Qualitative Study of Patients Receiving Immune Checkpoint Inhibitors for Melanoma or Lung Cancer and Their Caregivers. *Research Square* **2024**, rs.3.rs-4576328. https://doi.org/10.21203/rs.3.rs-4576328/v1

Author	Assessment Type	Health-Related Quality of Life (HRQoL) Measures	HRQoL Dimensions	Psychological Measures	Psychological Dimensions	Major Findings
Mancini J. [13]	Qualitative	Semi-structured Interviews	Leisure and daily activities; occupation and financial issues; physical well-being; relationship with healthcare professionals; relationship with family and friends; patient–caregiver relationship; relationships with institutional caregivers	Semi-structured Interviews	Psychological well-being: anxiety, fear, stress, sadness, depression, insecurity, and perceived injustice or unfairness; relationship with the patient: intimacy, communication, and emotional dynamics	In line with previous literature, the study found that caregiving was primarily associated with a significant psychological burden, even in cases involving patients with favorable prognoses, while serious physical consequences were rare. The findings highlight the need for a standardized assessment of caregivers' quality of life, supplemented by specific modules for cancer and relationships.
Weaver R. [16]	Qualitative	Semi-structured Interviews	Support with Medical Aspects of Caregiving: Medical tasks and care-related support; Need for Information about the Patient: Access to patient-related information; Financial Impact: Economic burden of caregiving	Semi-structured Interviews	Psychological Support for Caregivers and Family: Emotional and psychological support needs of caregivers and family members	Caregivers of sarcoma patients report that the patients' needs, which also affect them, are not being met in several areas: medical, informational, psychosocial and financial. Many caregivers have experienced psychological distress but have struggled to seek support, prioritising the needs of their patients, which has had a negative impact on their quality of life and ability to provide care. It is therefore necessary to develop support programmes tailored to caregivers, including financial assistance and support groups.
Fox J.A. [27]	Qualitative	-	-	Semi-structured Interviews (Grounded Theory)	Psychological dimensions: hope-related expectations, treatment continuation despite uncertain benefit, unmet informational needs, delayed integration of palliative care, and lack of end-of-life planning	The investigation reveals that care providers encounter a variety of difficulties, especially when it comes to acquiring clear infomrations from medical practitioners, for example during the transition to end-of-life care. Several factors contribute to caregivers' feelings of overwhelm and stress, including understanding complex prognostic data, navigating the debate between palliative and continuing care, and a lack of preparation for the end of life of the person they are caring for. These findings highlight the ongoing need for information, psychosocial support and preparation, and emphasise the importance of educational initiatives and improved communication between doctors, patients and caregivers to alleviate the burden on caregivers.
Thompson J.R. [21]	Mixed-Methods	European Organisation for Research and Treatment of Cancer Quality of Life Questionnaire (EORTC QLQ-C30); Supportive Care Needs Survey Partners&Caregiver (SCNS-P&C)	Caregiver quality of life: healthcare-related needs, work and social functioning, and global health	Depression, Anxiety and Stress Scale (DASS-21)	Psychological distress: anxiety, depression, stress, and distress related to unmet information needs	The study revealed that Australian melanoma patients and their caregivers have significant unmet psychological and emotional needs. The findings highlight the need for a standardized assessment of caregivers' quality of life, supplemented by specific modules on cancer and relationships. Focusing on these aspects would help facilitate the resolution of the issues identified.
Marshall-McKenna R. [24]	Mixed-Methods	Online Survey	Pervasive Uncertainty: Widespread and ongoing uncertainty across care trajectories	Online Survey	Psychological Needs: Need for psychological support; Relationship with Institutions and Information Needs: Interaction with institutions and access to information.	The analysis conducted by the authors shows that cancer survivors and their family caregivers report dissatisfaction with the lack of clear information and ongoing support throughout the cancer journey. Given these gaps, follow-up services and targeted psychological support are essential to address these shortcomings.
Makady A. [23]	Quantitative	Qualitative Interviews	Family and Social Relationships; Emotional Burden: Fear and concerns.	/	/	This study explored the feasibility of using social media to assess the perspectives of patients and their caregivers on health-related quality of life (HRQoL). The research found that caregivers prioritized the ability to cope with manageable adverse events, to be capable, and to be free from pain, while patients prioritized family, emphasizing the importance of leading a normal life and enjoying life. Some also emphasized the importance of not neglecting career-related aspects.
Johansen S. [9]	Quantitative	Caregiver Reaction Assessment (CRA); Medical Outcomes Study Social Support Survey (MOS-SSS); General Self-Efficacy Scale (GSES); General Sleep Disturbance Scale (GSDS)	Fatigue; Sleep Disturbance; Symptom Distress	Caregiver Reaction Assessment (CRA);<br>Lee Fatigue Scale (LFS); Center for Epidemiologic Studies Depression Scale (CES-D)	Psychological and social outcomes: fatigue, depression, sense of self-efficacy, stress and emotional distress, perceived social support	The research found that a significant burden on caregivers is associated with depression, fatigue, sleep disturbances, low self-esteem and limited social support in patients. The survey found that female patients and caregivers experienced a greater burden than men. These findings emphasise the importance of including caregivers in the cancer care pathway.
Milne D. [14]	Qualitative	Qualitative Interviews	Economic and physical burden: financial toxicity and fatigue	Qualitative Interviews	Treatment-related uncertainty and associated anxiety	This research focuses on patients receiving immunotherapy for stage IV melanoma and their caregivers, who report experiencing a reduced quality of life due to treatment-related toxicities, stress, financial hardship and fatigue. Key challenges include uncertainty and an increased burden of caregiving responsibilities and side effects. The findings highlight the need for comprehensive preparation, clear information and rapid access to knowledgeable healthcare professionals to support patients and their caregivers.
Shilling V. [18]	Qualitative	Qualitative Interviews	Job and financial implications: concerns related to employment, loss of earnings, and perceived financial position<br>Implications for the future: changes in outlook, realigning priorities, life on hold, opportunities lost, and inability to plan for the future	Qualitative Interviews	Managing uncertainty: control, preservation of or return to normality, hope, and mindset<br>Relationships and communication: patient–caregiver relationship and communication, prevalence of cancer-related conversations, and family dynamics	Patients and caregivers faced significant uncertainty about the future, leading to loss of control over work, finances, family, and retirement. Coping focused on maintaining “normality,” with family well-being prioritized by patients and caregivers feeling their lives “on hold.” Impact varied by age and closeness to the patient, highlighting the need for open communication and targeted support.
Aguiar-Ibanez R. [15]	Qualitative	Online Survey; Work Productivity and Activity Impairment: Caregiver (WPAI: CG)	Work productivity and activity impairment	Online Survey	/	Cancer recurrence has a substantial negative impact on work productivity, employment stability, and financial well-being for both patients and caregivers. These challenges contribute to increased stress, limit the ability to participate in daily activities, and exacerbate the overall burden of caregiving, highlighting the need for targeted support and interventions to mitigate these effects.
Boulanger M.C. [22]	Qualitative	Qualitative Interviews	Disruption of family and occupational roles	Qualitative Interviews	Hope and prognostic uncertainty; overwhelming disappointment among patients without long-term response; conflicting preferences for receiving prognostic information; chronic stress related to ongoing prognostic uncertainty and constant vigilance; perceived lack of control over the course of treatment and the patient’s prognosis	Optimistic expectations were often influenced by oncology teams, but uncertainty and unpredictable long-term treatment responses caused emotional distress and disappointment. Patients and caregivers had differing preferences for prognostic information, underscoring the need for personalized communication strategies.
Papanikolaou E.S. [19]	Quantitative	Family Dermatology Life Quality Index (FDLQI)	Time dedicated to caregiving; impact on work or studies; social activities and leisure time; personal relationships; daily activities; financial aspects; sleep and rest; overall impact on quality of life	Family Dermatology Life Quality Index (FDLQI)	Emotional and psychological well-being; self-perception in the caregiver role	Emotional distress was identified as the primary contributor to caregiver burden on the FDLQI. Burden was higher for son/daughter caregivers and increased with patient age and time since diagnosis, whereas caregiver sex, age, and educational level showed no significant effect.

Muliira J.K. [17]	Quantitative	/	/	Caregiver Burden Scale (CBS)	Psychological dimensions: emotional and psychological well-being; self-perceived role and identity as a caregiver	Caregivers in low-income settings experience high, multidimensional burden, including physical, emotional, and social impacts. Key interventions to alleviate this burden include emotional support, training, and practical assistance such as dedicated nursing care.
Tan J.D. [26]	Qualitative	Semi-structured Phone Interviews	Treatment impact: disruption of daily life, including occupational, social, and family roles; financial impact; caregiver role devaluation	Semi-structured Phone Interviews	Emotional distress; feelings of isolation, helplessness, and lack of support from healthcare systems; coping strategies, including adaptive (support, helpful thinking, meaning-making) and maladaptive (avoidance, suppression)	Patients and caregivers reported significant emotional distress across all disease phases, including shock, anxiety, fear, sadness, and frustration. Caregivers assumed new roles during treatment, leading to feelings of inadequacy, guilt, and being overwhelmed, with some experiencing devaluation of their own struggles. Coping strategies varied by disease phase. Findings highlight the need for routine psychological screening, enhanced communication, and targeted supportive care for both patients and caregivers.
Yabroff K.R. [20]	Quantitative	Survey	Time costs related to caregiving; financial impact	Survey	Emotional support and instrumental support (providing practical help and assistance)	Caregiving for cancer patients imposes substantial time demands, averaging 8.3 hours per day over 13.7 months, with the highest burden for patients with distant disease. About half of caregivers provided emotional, instrumental, tangible, or medical support, highlighting the significant time-related burden and costs in the first two years after diagnosis.
Kim Y. [25]	Qualitative	FACIT-Sp	Spiritual Well-Being	Profile od Mood States (POMS-SF); Pearlin Stress Scale;	Psychological dimensions: caregiving stress; caregiver spirituality; caregiver psychological distress	Caregivers of survivors with nongender-specific cancers experienced higher psychological distress than those caring for gender-specific cancers. Increased caregiving stress and lower spirituality were linked to greater distress, whereas higher spirituality mitigated stress effects, emphasizing the influence of cancer type and personal spiritual resources on caregiver adjustment.

Database	Date of last search	Search Strategy	Results (N)
PubMed	21/01/2025	((sarcoma[Title/Abstract]) AND (caregiver[Title/Abstract])) OR ((melanoma[Title/Abstract]) AND (caregiver[Title/Abstract]))<br><br>((sarcoma[Title/Abstract]) AND (spouse[Title/Abstract])) OR ((melanoma[Title/Abstract]) AND (spouse[Title/Abstract]))<br><br>((sarcoma[Title/Abstract]) AND (informal caregiver[Title/Abstract])) OR ((melanoma[Title/Abstract]) AND (informal caregiver[Title/Abstract]))<br><br>((sarcoma[Title/Abstract]) AND (family caregiver[Title/Abstract])) OR ((melanoma[Title/Abstract]) AND (family caregiver[Title/Abstract]))<br>**Tot**	41<br><br>37<br><br>2<br><br>0<br><br>**80**
Embase	21/01/2025	1# sarcoma:ab,ti AND 'caregiver':ab,ti OR (melanoma:ab,ti AND 'caregiver':ab,ti)<br>2# sarcoma:ab,ti AND 'spouse':ab,ti OR (melanoma:ab,ti AND 'spouse':ab,ti)<br>3# sarcoma:ab,ti AND 'informal caregiver':ab,ti OR (melanoma:ab,ti AND 'informal caregiver':ab,ti)<br>4# sarcoma:ab,ti AND 'family caregiver':ab,ti OR (melanoma:ab,ti AND 'family caregiver':ab,ti)<br>**Tot**	98<br>99<br>6<br>1<br>**204**
PsychINFO	21/01/2025	XB (sarcoma) AND XB (caregiver)<br>XB (melanoma) AND XB (caregiver)<br>XB (sarcoma) AND XB (spouse)<br>XB (melanoma) AND XB (spouse)<br>XB (sarcoma) AND XB (informal caregiver)<br>XB (melanoma) AND XB (informal caregiver)<br>XB (melanoma) AND XB (family caregiver)<br>XB (sarcoma) AND XB (family caregiver)<br>**Tot**	7<br>15<br>2<br>9<br>1<br>3<br>3<br>1<br>**41**
**Total**			325

DATA EXTRACTION FORM

**STUDY DETAILS**	
Authors	
Title	
Type of publication	● Article<br>● Book chapter
Year of publication	
Type of study	● Randomized Controlled Trials<br>● Quasi-Experimental Studies<br>● Cohort Studies<br>● Case-Control Studies<br>● Cross-Sectional Studies<br>● Case Reports/Series<br>● Qualitative Studies
**SAMPLE CHARACTERISTICS**	
**N**	
Age	
Gender	
Relationship	
Country	
Type of cancer	
Phase of cancer	
Educational level (when available)	
**MEASURES**	
Type of assessment	● Qualitative<br>● Quantitative
Qol tools (for caregiver)	
Qol dimensions measured	
Psychological tools (for caregiver)	
Psychological dimension measured	
**MAJOR FINDINGS**	

Author	Title	Type of pub	Year	Study design	Country	Number of patients (caregivers)	Age of patients (mean, SD or range)	Gender of patients	Relationship	Type of cancer	Stage of cancer
Mancini J. [13]	Quality of life in a heterogeneous sample of caregivers of cancer patients: An in-depth interview study.	Article	2011	Qualitative Research	France	77 (17 melanoma)	48,1 (18-81) (melanoma 55.8 (32-70))	F:49 M:28 Melanoma F:8 M:9	2 parent, 12 spouse, 2 child, 1 friend	breast cancer,<br>melanoma, paediatric haematology	II, III (for melanoma)
Weaver R. [16]	The unmet needs of carers of patients diagnosed with sarcoma: A qualitative study.	Article	2021	Qualitative Research	Australia	33	51 (range 22-66)	F:26 M:12	15 mother, 4 father, 11 spouse, 2 brother, 1 daughter	sarcoma	-
Fox J.A. [27]	Palliative care in the context of immune and targeted therapies: A qualitative study of bereaved carers' experiences in metastatic melanoma.	Article	2020	Qualitative Research	Australia	20	30 to 39 years 1 (5%)<br>40 to 49 years 4 (20%)<br>50 to 59 years 3 (15%)<br>60 to 69 years 9 (45%)<br>70 to 79 years 3 (15%)	F:16 M:4	16 partner, 2 sibling, 2 child	metastatic melanoma	-
Thompson J.R. [21]	Supportive care needs in Australian melanoma patients and caregivers: results from a quantitative cross-sectional survey.	Article	2023	Quantitative Cross-sectional Survey	Australia	37	55 (12)	F:32 M:5	29 partner, 8 immediate family member	melanoma	early and advanced stage
Marshall-McKenna R. [24]	A multinational investigation of healthcare needs, preferences, and expectations in supportive cancer care: Co-creating the LifeChamps digital platform.	Article	2022	Descriptive, Cross-sectional, Multi-method study.	Greece, Spain, Sweden, United Kingdom	23	50,3 (14,8)	F:9 M:4	10 daughter, 8 spouse/partner, 3 other, 1 sister in law, 1 son	breast cancer, prostate cancer, melanoma	cancer survivor
Makady A. [23]	Social media as a tool for assessing patient perspectives on quality of life in metastatic melanoma: A feasibility study.	Article	2018	Survey	Belgium, France, Netherlands, Romania, United Kingdom, other	17	<40 (6%)<br>40 to 49 years (12%)<br>50 to 59 years (24%)<br>60 to 69 years (29%)<br>70 to 79 years (24%)<br>80+ (6%)	F:17 M:9	-	melanoma	all stage
Johansen S. [9]	The effect of cancer patients' and their family caregivers' physical and emotional symptoms on caregiver burden.	Article	2018	Cross-sectional quantitative research	Norway	281 (42 head neck and skin)	≤50 (96)<br>> 50 (185)	F:149 M:132	227 spouse/partner, 45 family member, 9 other family member	breast, prostate, melanoma, myelomatose, lymphoma, head-neck cancers	-
Milne D. [14]	Exploring the experiences of people treated with immunotherapies for advanced melanoma and those caring for them: 'Real-world' data.	Article	2020	Qualitative Research, Cross-sectional	Australia	9	49 (range 32-65)	F:8 M:1	-	melanoma	IV
Shilling V. [18]	The pervasive nature of uncertainty—A qualitative study of patients with advanced cancer and their informal caregivers.	Article	2017	Qualitative Research	United Kingdom	8	53 (36-70)	F:5 M:3	spouse/partner	ovarian, melanoma, lung cancer	III, IV
Aguiar-Ibanez R. [15]	Impact of recurrence on employment, finances, and productivity for early-stage cancer patients and caregivers: US survey	Article	2024	Cross-sectional, Non-interventional, Online survey	United States of America	100 (17 melanoma)	51.7	F:71 M:28 Non binary:1	48 spouse/significant other, 24 son/daughter, 10 parent, 9 friend/neighbour, 9 sibling	bladder, gastric, head and neck, non–small cell lung,<br>renal cell, triple-negative breast cancers, melanoma	-
Boulanger M.C. [22]	Patient and caregiver experience with the hope and prognostic uncertainty of immunotherapy: A qualitative study	Article	2024	Qualitative Research	United States of America	10 (7 melanoma)	77 (31-80)	F:5 M:4 Missing: 1	8 Spouse, 1 sibling, 1 other family member	melanoma, NSCLC	III, IV
Papanikolaou E.S. [19]	Quality of life in caregivers of melanoma patients	Article	2022	-	Italy	120	<40 (36,7%)<br>40-54 (40,8%)<br>> 55 (22,5%)	F:51 M:69	50 son/daughter, 23 partner, 27 brother/sister, 20 other	melanoma	all stage
Muliira J.K. [17]	Roles of family caregivers and perceived burden when caring for hospitalized adult cancer patients: Perspective from a low-income country	Article	2018	Cross sectional, Descriptive design	Africa (Uganda)	168	36 (12.7)	F:128 M:40	46 spouse, 122 not spouse	kaposi's sarcoma, prostate carcinoma, leukemia, pancreatic cancer, esophageal cancer, bone cancer, seminoma, hepatocarcinoma, colorectal cancer, breast cancer	-
Tan J.D. [26]	A qualitative assessment of psychosocial impact, coping and adjustment in high-risk melanoma patients and caregivers	Article	2014	Qualitative Research	Australia	14	57 (11)	F:11 M:3	8 partner, 1 parent, 1 child, 2 friend, 2 other	melanoma	III
Yabroff K.R. [20]	Time costs associated with informal caregiving for cancer survivors	Article	2009	Qualitative research	United States of America	688 (73 bladder, skin and uterine)	<24 (1.5%)<br>25-34 (5.8%)<br>35-44 (12.4%)<br>45-54 (32.4%)<br>55-64 (26.2%)<br><65 (19%)<br>Missing data n=19 (2.8%)	F:450 M:238	451 spouse/partner, 111 child/child-in-law, 29 parent, 57 sibling, 26 friend, 14 other	bladder, breast, colorectal, kidney, lung, melanoma of the skin, ovarian, prostate, or uterine cancer, non-Hodgkins lymphoma (NHL)	-
Kim Y. [25]	Psychological distress of female cancer caregivers: Effects of type of cancer and caregivers' spirituality	Article	2007	Qualitative research	United States of America	1635 (7 melanoma)	48.35 (14.52)	F:1068 M:567	28 mother, 60 sister, 110 daughter, 21 friend, 8 daughter-in-law,<br>6 other in-law, 7 partner, 12 other (just female)	breast, kidney, lung, non-Hodgkin's lymphoma, melanoma, ovarian cancer.	-

Assessment tools	Construct assessed	Domains/Subscales	QoL or Psychological Domains	Number of items	Response format	Mode of administration	Score range	Interpretation	Target population	Validity (Cronbach's alpha)	Psychometric informations in caregiver populations	Studies using the instrument included in the revision paper	Population on which the instrument was administered	Clinical context in wich instrument was used (disease)	Clinical context in wich instrument was used (stage of disease)	Validation article reference used in the articles included in the revision paper
European Organisation for Research and Treatment of Cancer Quality of Life Questionnaire (EORTC QLQ-C30)	Helath Related Quality of Life	5 functional scales (physical, role, cognitive, emotional, social), 3 general symptom scales (fatigue, nausea/vomiting, pain), 6 single symptom scales (dyspnea, insomnia, appetite loss, constipation, diarrhoea, financial impact)	QoL	30	4-point likert scale (1-4) for functional and symptom scales; 6-point scale (1-7) for global health	Self-administered questionnaire	0–100 per scale	Higher scores indicate better functioning for functional scales and worse symptoms for symptom scales; global QoL higher indicate better QoL	Adult cancer patients	>0.70	Not validated on caregivers population	Thompson J. R. [21]	Caregivers	Melanoma	Early and advanced stage	Aaronson, N. K.; Ahmedzai, S.; Bergman, B.; Bullinger, M.; Cull, A.; Duez, N. J.; Filiberti, A.; Flechtner, H.; Fleishman, S. B.; de Haes, J. C. J. M.; Kaasa, S.; Klee, M.; Osoba, D.; Razavi, D.; Rofe, P. B.; Schraub, S.; Sneeuw, K.; Sullivan, M.; Takeda, F. The European Organization for Research and Treatment of Cancer QLQ-C30: A quality-of-life instrument for use in international clinical trials in oncology. *J. Natl. Cancer Inst.* **1993**, 85(5), 365–376. doi:10.1093/jnci/85.5.365.
Supportive Care Needs Survey for Partners and Caregivers (SCNS-P&C)	Unmet supportive care needs of partners and caregivers	Health-care service needs<br>Psychological and emotional needs<br>Work and social needs<br>Information needs	Both	44	5-point Likert scale (1-5)	Self-administered questionnaire	1–5 per item	Higher scores indicate greater unmet needs	Partners and caregivers of cancer survivors	0.88 – 0.94	Validated on caregivers population	Thompson J. R. [21]	Caregivers	Melanoma	Various stages	Girgis, A.; Lambert, S.; Lecathelinais, C. The supportive care needs survey for partners and caregivers of cancer survivors: development and psychometric evaluation. *Psycho-Oncology* **2011**, 20(4), 387–393. doi: 10.1002/pon.1740.
Depression, Anxiety and Stress Scale - 21 Items (DASS-21)	Depression, anxiety, and stress	Depression, anxiety, stress	Psychological Domains	21	4-point Likert scale (0-3)	Self-administered questionnaire	Scores summed per subscale	Higher scores indicate greater symptom severity	General/Clinical population	0.88 depression, 0-82 anxiety, 0.92 stress, 0.93 total scale	Not validated on caregivers population	Thompson J. R. [21]	Caregivers	Melanoma	Various stages	Henry, J. D.; Crawford, J. R. The short-form version of the Depression Anxiety Stress Scales (DASS-21): construct validity and normative data in a large non-clinical sample. *Br. J. Clin. Psychol.* **2005**, 44(Pt 2), 227–239. doi:10.1348/014466505X29657.
Caregiver Reaction Assessment (CRA)	Caregiving burden	Self Esteem, Lack of Family Support, Impact on Finances, Impact on Daily Schedule, Impact on Health	Psychological Domains	24	5-point Likert scale (1-5)	Self-administered questionnaire	Scores summed per subscale	Higher scores indicate greater burden except for Self Esteem (higher = positive)	General/Clinical population	0.57-0-85, 0.74 total	Validated on caregivers population	Johansen S. [9]	Caregivers	Melanoma	Stage IV	Grov, E. K.; Fosså, S. D.; Tønnessen, A.; Dahl, A. A. The caregiver reaction assessment: psychometrics, and temporal stability in primary caregivers of Norwegian cancer patients in late palliative phase. *Psycho‐Oncology* **2006**, 15(6), 517–527. doi:10.1002/pon.987.
Profile of Mood States - Short Form (POMS-SF)	Mood	Tension or Anxiety, Depression or Dejection, Anger or Hostility, Vigor or Activity, Fatigue or Inertia, Confusion or Bewilderment	Psychological Domains	37	5-point Likert scale (0-4)	Self-administered questionnaire	Scores summed per subscale	Higher scores indicate greater intensity of mood state	General population	0.85-0.95	Validated on caregivers population	Kim Y. [25]	Caregivers	Breast, kidney, lung, non-Hodgkin' slymphoma, melanoma, ovarian cancer.	-	McNair, D. M.; Lorr, M.; Droppleman, L. F. Profile of Mood States, Revised ed.; EdITS/Educational and Industrial Testing Service: San Diego, CA, 1992.
Family Dermatology Life Quality Index (FDLQI)	Impact of skin diseases on family quality of life	-	QoL	10	4-point Likert scale (0-3)	Self-administered questionnaire	0–30 total	Higher scores indicate greater negative impact on family members' quality of life	Family members of dermatology patients	0.88	Validated on caregivers population	Papanikolaou E.S. [19]	Caregivers	Melanoma	All stages	Basra, M. K. A.; Sue‐Ho, R.; Finlay, A. Y. The Family Dermatology Life Quality Index: measuring the secondary impact of skin disease. *Br. J. Dermatol.* **2007**, 156(3), 528–538. doi:10.1111/j.1365‐2133.2006.07617.x.
Medical Outcomes Study Social Support Survey (MOS-SSS)	Perceived social support	Emotional/Informational support<br>Tangible support<br>Affectionate support<br>Positive social interaction support	Psychological Domains	19	5-point Likert scale (1-5)	Self-administered questionnaire	0–100 per subscale	Higher scores indicate greater perceived support	Patients with chronic illnesses	0.91	Not validated on caregivers population	Johansen S. [9]	Caregivers	Breast, prostate, melanoma, myelomatose, lymphoma, headneckcancers	-	Sherbourne, C. D.; Stewart, A. L. The MOS social support survey. *Soc. Sci. Med.* **1991**, 32(6), 705–714. doi:10.1016/0277‐9536(91)90150‐B.
General Self Efficacy Scale (GSES)	General self-efficacy	-	Psychological Domains	10	4-point likert scale (1-4)	Self-administered questionnaire	10-40 total	Higher scores indicate more self-efficacy	General population	0.76 – 0.90	Not validated on caregivers population	Johansen S. [9]	Caregivers	Breast, prostate, melanoma, myelomatose, lymphoma, headneckcancers	-	Schwarzer, R.; Jerusalem, M.; Weinman, J.; Wright, S.; Johnston, M. Generalized Self‐Efficacy Scale. In Measures in Health Psychology: A User's Portfolio. Causal and Control Beliefs; NFER-NELSON: Windsor, UK, 1995.
General Sleep Disturbance Scale (GSDS)	Incidence and nature of sleep disturbance	Quality of sleep, Quantity of sleep, Sleep onset latency, Midsleep awakenings, Early awakenings, Medications for sleep, Excessive daytime sleepiness	QoL	21	8-point Likert-type scale (0-7)	Self-administered questionnaire	0-147 total	Higher total and subscale scores indicated higher levels of sleep disturbance. Subscales scores of ≥ 3 and a GSDS total score of ≥ 43 indicates a significant level of sleep disturbance	General population	0.79	Validated on caregivers population	Johansen S. [9]	Caregivers	Breast, prostate, melanoma, myelomatose, lymphoma, headneckcancers	-	Carney, S.; Koetters, T.; Cho, M.; West, C.; Paul, S. M.; Dunn, L.; Aouizerat, B. E.; Dodd, M.; Cooper, B.; Lee, K.; Wara, W.; Swift, P.; Miaskowski, C. Differences in sleep disturbance parameters between oncology outpatients and their family caregivers. *J. Clin. Oncol.* **2011**, 29 (8), 1001–1006. doi:10.1200/JCO.2010.30.9104.
Lee Fatigue Scale (LFS)	Fatigue severity	Fatigue subscale, Energy subscale	QoL	18	Visual Analog Scale	Self-administered questionnaire	0-30, 0-50, 0-130	Higher scores indicate higher levels of perceived fatigue and energy	General/Clinical population	0.94 – 0.96	Validated on caregivers population	Johansen S. [9]	Caregivers	Breast, prostate, melanoma, myelomatose, lymphoma, headneckcancers	-	Lee, K. A.; Hicks, G.; Nino‐Murcia, G. Validity and reliability of a scale to assess fatigue. *Psychiatry Res.* **1991**, 36(3), 291–298. doi:10.1016/0165‐1781(91)90027‐M.
Center for Epidemiologic Studies Depression Scale (CES-D)	Presence and severity of depressive symptoms	-	Psychological Domains	20	4-point Likert scale (0-3)	Self-administered questionnaire	0-60	Higher scores indicate greater depressive symptoms (cut-off = 16)	General population	<0.85	Validated on caregivers population	Johansen S. [9]	Caregivers	Breast, prostate, melanoma, myelomatose, lymphoma, headneckcancers	-	Radloff, L.S. The CES-D Scale: A Self-Report Depression Scale for Research in the General Population. *Appl. Psychol. Meas.* **1977**, 1, 385–401. https://doi.org/10.1177/014662167700100306
Caregiver Burden Scale (CBS)	Caregiver burden	General strain, Isolation, Disappointment, Emotional Involment, Environment	Both	22	5-point Likert scale (0-4)	Self-administered questionnaire	0-88	Higher scores indicate greater caregiver burden (0-20 = little/no burden, 21-40 = mild/moderate burden, 41-60 = moderate/severe burden, 61-88 = severe burden)	Caregiver population	0.70 – 0.87 (except for environment)	Validated on caregivers population	Muliira J.K. [17]	Caregivers	Kaposi's sarcoma, prostate carcinoma, leukemia, pancreatic cancer, esophageal cancer, bone cancer, seminoma, hepatocarcinoma, colorectal cancer,breast cancer	-	Elmståhl, S.; Malmberg, B.; Annerstedt, L. Caregiver's burden of patients 3 years after stroke assessed by a novel caregiver burden scale. *Arch. Phys. Med. Rehabil.* **1996**, 77, 177–182. doi:10.1016/S0003-9993(96)90164-1.
Pearlin Role Overload Measure (Pearlin ROM)	Stress	-	Psychological Domains	4	4-point likert scale (1-4)	Self-administered questionnaire	4-16	Higher scores indicate greater overload	Caregiver population	0.80	Validated on caregivers population	Kim Y. [25]	Caregivers	Breast, kidney, lung, non-Hodgkin' slymphoma, melanoma, ovarian cancer.	-	Pearlin, L. I.; Mullan, J. T.; Semple, S. J.; Skaff, M. M. Caregiving and the stress process: An overview of concepts and their measures. *Gerontologist* **1990**, 30, 583–594. doi:10.1093/geront/30.5.583.
FACIT-Sp12	Spritual Well-being	Spritual Well-being, Meaning, Peace, Faith	QoL	12	5-point Likert scale (0-4)	Self-administered questionnaire	0-48	Higher scores better QoL Spritual Well-being	Clinical Population	0.89	Validated on caregivers population	Kim Y. [25]	Caregivers	Breast, kidney, lung, non-Hodgkin' slymphoma, melanoma, ovarian cancer.	-	Peterman, A. H.; Fitchett, G.; Brady, M. J.; Hernandez, L.; Cella, D. Measuring spiritual well-being in people with cancer: the Functional Assessment of Chronic Illness Therapy–Spiritual Well-Being Scale (FACIT-Sp). *Ann. Behav. Med.* **2002**, 24, 49–58. doi:10.1207/S15324796ABM2401_06.
Work Productivity and Activity Impairment: Caregiver (WPAI:CG)	Work and activity impairment	Absenteism, Presenteism, Overall Work Impairment, Activity Impairment	QoL	6	1 item yes/no, 3 open item, 2 visual analog scale (0-10)	Self-administered questionnaire	0-100%	Higher scores indicate less interference	Patients with health condition	<0.70	Validated on caregivers population	Aguiar-Ibanez R. [15]	Caregivers	Bladder, gastric, head and neck, non–small cell lung,renal cell, triple-negative breastcancers, melanoma	-	Reilly, M. C.; Zbrozek, A. S.; Dukes, E. M. The validity and reproducibility of a work productivity and activity impairment instrument. *Pharmacoeconomics* **1993**, 4(5), 353–365. doi:10.2165/00019053-199304050-00006.

Preferred Reporting Items for Systematic reviews and Meta-Analyses extension for Scoping Reviews (PRISMA-ScR) Checklist

SECTION	ITEM	PRISMA-ScR CHECKLIST ITEM	REPORTED ON PAGE #
**TITLE**			
Title	1	Identify the report as a scoping review.	1
**ABSTRACT**			
Structured summary	2	Provide a structured summary that includes (as applicable): background, objectives, eligibility criteria, sources of evidence, charting methods, results, and conclusions that relate to the review questions and objectives.	1-2
**INTRODUCTION**			
Rationale	3	Describe the rationale for the review in the context of what is already known. Explain why the review questions/objectives lend themselves to a scoping review approach.	2-3
Objectives	4	Provide an explicit statement of the questions and objectives being addressed with reference to their key elements (e.g., population or participants, concepts, and context) or other relevant key elements used to conceptualize the review questions and/or objectives.	3
**METHODS**			
Protocol and registration	5	Indicate whether a review protocol exists; state if and where it can be accessed (e.g., a Web address); and if available, provide registration information, including the registration number.	3, Not regist.
Eligibility criteria	6	Specify characteristics of the sources of evidence used as eligibility criteria (e.g., years considered, language, and publication status), and provide a rationale.	3-4
Information sources*	7	Describe all information sources in the search (e.g., databases with dates of coverage and contact with authors to identify additional sources), as well as the date the most recent search was executed.	4
Search	8	Present the full electronic search strategy for at least 1 database, including any limits used, such that it could be repeated.	4, Tab. S2.
Selection of sources of evidence†	9	State the process for selecting sources of evidence (i.e., screening and eligibility) included in the scoping review.	4-5
Data charting process‡	10	Describe the methods of charting data from the included sources of evidence (e.g., calibrated forms or forms that have been tested by the team before their use, and whether data charting was done independently or in duplicate) and any processes for obtaining and confirming data from investigators.	5-6, Tab. S3
Data items	11	List and define all variables for which data were sought and any assumptions and simplifications made.	5-6
Critical appraisal of individual sources of evidence§	12	If done, provide a rationale for conducting a critical appraisal of included sources of evidence; describe the methods used and how this information was used in any data synthesis (if appropriate).	Not applicable
Synthesis of results	13	Describe the methods of handling and summarizing the data that were charted.	6

St. Michael's
Inspired Care.
Inspiring Science.

SECTION	ITEM	PRISMA-ScR CHECKLIST ITEM	REPORTED ON PAGE #
**RESULTS**			
Selection of sources of evidence	14	Give numbers of sources of evidence screened, assessed for eligibility, and included in the review, with reasons for exclusions at each stage, ideally using a flow diagram.	5, Tab. 1
Characteristics of sources of evidence	15	For each source of evidence, present characteristics for which data were charted and provide the citations.	5-6
Critical appraisal within sources of evidence	16	If done, present data on critical appraisal of included sources of evidence (see item 12).	Not applicable
Results of individual sources of evidence	17	For each included source of evidence, present the relevant data that were charted that relate to the review questions and objectives.	6-13
Synthesis of results	18	Summarize and/or present the charting results as they relate to the review questions and objectives.	6-14
**DISCUSSION**			
Summary of evidence	19	Summarize the main results (including an overview of concepts, themes, and types of evidence available), link to the review questions and objectives, and consider the relevance to key groups.	14-18
Limitations	20	Discuss the limitations of the scoping review process.	17-18
Conclusions	21	Provide a general interpretation of the results with respect to the review questions and objectives, as well as potential implications and/or next steps.	18-19
**FUNDING**			
Funding	22	Describe sources of funding for the included sources of evidence, as well as sources of funding for the scoping review. Describe the role of the funders of the scoping review.	19

JBI = Joanna Briggs Institute; PRISMA-ScR = Preferred Reporting Items for Systematic reviews and Meta-Analyses extension for Scoping Reviews.
* Where *sources of evidence* (see second footnote) are compiled from, such as bibliographic databases, social media platforms, and Web sites.
† A more inclusive/heterogeneous term used to account for the different types of evidence or data sources (e.g., quantitative and/or qualitative research, expert opinion, and policy documents) that may be eligible in a scoping review as opposed to only studies. This is not to be confused with *information sources* (see first footnote).
‡ The frameworks by Arksey and O'Malley (6) and Levac and colleagues (7) and the JBI guidance (4, 5) refer to the process of data extraction in a scoping review as data charting.
§ The process of systematically examining research evidence to assess its validity, results, and relevance before using it to inform a decision. This term is used for items 12 and 19 instead of "risk of bias" (which is more applicable to systematic reviews of interventions) to include and acknowledge the various sources of evidence that may be used in a scoping review (e.g., quantitative and/or qualitative research, expert opinion, and policy document).

From: Tricco AC, Lillie E, Zarin W, O'Brien KK, Colquhoun H, Levac D, et al. PRISMA Extension for Scoping Reviews (PRISMA-ScR): Checklist and Explanation. Ann Intern Med. ;169:467–473. doi: 10.7326/M18-0850
